# Expanding growers' choice of plant disease management options can promote suboptimal social outcomes

**DOI:** 10.1111/ppa.13705

**Published:** 2023-02-06

**Authors:** Rachel E. Murray‐Watson, Nik J. Cunniffe

**Affiliations:** ^1^ Department of Plant Sciences University of Cambridge Cambridge UK

**Keywords:** behavioural model, epidemiological model, Gini coefficient, Pareto optimization, tomato yellow leaf curl virus (TYLCV)

## Abstract

Previous models of growers' decision‐making during epidemics have unrealistically limited disease management choices to just two options. Here, we expand previous game‐theoretic models of grower decision‐making to include three control options: a crop that is tolerant, resistant or susceptible to disease. Using tomato yellow leaf curl virus (TYLCV) as a case study, we investigate how growers can be incentivized to use different control options to achieve socially optimal outcomes. To do this, we consider the efforts of a ‘social planner’ who moderates the price of crops. We find that subsidizing a tolerant crop costs the social planner more in subsidies, as its use encourages selfishness and widespread adoption. Subsidizing a resistant crop, however, provides widespread benefits by reducing the prevalence of disease across the community of growers, including those that do not control, reducing the number of subsidies required from the social planner. We then use Gini coefficients to measure equitability of each subsidization scheme. This study highlights how grower behaviour can be altered using crop subsidies to promote socially optimal outcomes during epidemics.

## Graphical abstract

Growers face a range of decisions when choosing how to control for plant diseases, but their management decisions may have negative impacts for other growers.

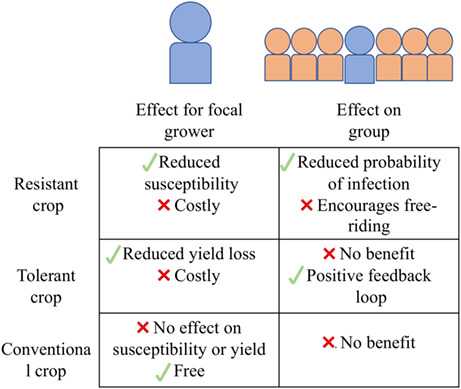

## 1 INTRODUCTION

Those tasked with managing epidemics typically have limited resources, which they must distribute efficiently. This often incurs a trade‐off involving multiple parties with conflicting objectives, and how these trade‐offs are managed will depend on the relative priority of each objective. A well‐known example of such a trade‐off for disease management occurs during vaccination campaigns (e.g., Gavish & Katriel, 2022). Members of the public often wish to minimize their infection risk, while governments may want to optimize the allocation of vaccines to protect the most vulnerable (Gavish & Katriel, 2022). Similarly, in the context of plant epidemics, growers faced with crop disease will want to maximize profits, but with this comes potential environmental damage (Zaffaroni & Bevacqua, 2022) or reduced profitability when there is no risk of infection (Vyska et al., 2016). The control strategy adopted by growers may also have negative consequences for others; Grogan and Goodhue (2012) found that when growers sprayed pesticides within their non‐citrus fields, the pesticides killed parasitic wasps that are the natural enemy of pests in neighbouring citrus fields. This intensified the reliance of citrus growers on pesticides, increasing their expenditure and environmental impact. These scenarios bear resemblance to the classical ‘tragedy of the commons’ (Hardin, 1968), where the self‐interested actions of individuals cause worse outcomes for others.

In economics, a 'social planner' is a decision‐maker whose goal is to balance the trade‐offs that result from a policy action, aiming to maximize the welfare across all parties (Sugden, 2013). Originally conceptualized for welfare economics (Sugden, 2013), the social planner's problem has been used in a range of settings, such as infectious disease management (Toxvaerd, 2019), wildlife conservation (Johannesen & Skonhoft, 2005) and supply chain management (Lee & Choi, 2021). A key aspect of the social planner is that they account for the externalities associated with certain actions. In economics, externalities are the consequences of an action that are felt by a third party not involved in the action (Gersovitz, 2014). These externalities are often ignored by individuals, who only care about their own outcome, but they are important when considering what is socially optimal.

The social planner's problem can be solved using Pareto optimality. Under a Pareto‐optimal solution, we cannot improve one party's objective without ensuring a worse outcome for another party (Luc, 2008). These solutions are efficient, as they optimize for each outcome, and non‐dominated, as, given the same constraints, they cannot be improved upon by any other solution. There may be many possible solutions for a given problem, which combine to give a Pareto front (a set of solutions with equally good outcomes). Pareto fronts have been calculated to visualize the trade‐offs involved in the recent coronavirus pandemic (Yousefpour et al., 2020), as well as to evaluate retrospectively responses to the 1918 influenza pandemic (Velde, 2022) and to understand better how to allocate healthcare equipment optimally during an epidemic (Donmez et al., 2022). In plant disease modelling, they have been used to investigate the trade‐offs between crop productivity and environmental impacts (Zaffaroni & Bevacqua, 2022). None of the solutions in the Pareto front is inherently better than any other, so the decision‐maker must decide which option to use based on additional information. Pareto optimality has some similarities to game theory; both are concerned with achieving the ‘best possible outcome’. However, while game theory is concerned with the optimal outcome of each individual player, the Pareto optimum is concerned with the optimum state of the entire system.

Here, we use Pareto fronts to study the social planner's problem in the context of a plant disease epidemic. The individuals in a population of growers can choose between different crop varieties that vary in their losses in yield if they become infected, their susceptibility to infection and/or their potential as a source of inoculum (i.e., disease can spread to surrounding fields). The behavioural component of the model is based on game theory, an economic tool used to study the strategic decision‐making undertaken by individuals when their choice of action strongly depends on the actions of others. Previous work that incorporated human behaviour into plant epidemic models has only allowed growers two options, ‘control’ or ‘not control’ (Bate et al., 2021; McQuaid et al., 2017; Milne et al., 2016, 2020; Murray‐Watson et al., 2022; Murray‐Watson & Cunniffe, 2022; Saikai et al., 2021), but such a narrow choice is unlikely, and in practice, growers will instead choose from a range of control options, each with different characteristics.

We adapt the game‐theoretic models described in Milne et al. (2016) and subsequent literature to allow growers an expanded set of strategies. We extend the model to go beyond the previous choice between ‘control’ and ‘not control’ to allow growers to choose between three crop types: two control options (disease‐resistant or disease‐tolerant crop) or an unimproved crop. Growers' decisions on which crop type to use will depend on their estimation of the profit they expect to earn over the next growing season if they were to use each crop type, which in turn depends on the prevalence of infection and the proportion of other growers using each control strategy. The models are based on ‘strategic adaptive’ expectations (Fenichel & Wang, 2013), where growers balance their knowledge of the current state of the system with their experience from the previous season. In particular, growers compare their profit from the preceding season with the expected profit over the next season for each of the strategies that they had not used (the ‘strategy vs. alternative’ models described in Murray‐Watson et al., 2022 and Murray‐Watson & Cunniffe, 2022). This requires growers to have ‘perfect information’ of the risk of infection of each crop variety, the control strategies of all other players and the profits for each outcome. It allows growers to balance sources of information, acting as ‘reflexive producers’ (Kaup, 2008).

Resistance and tolerance are the two main mechanisms of plant disease defence. Disease‐resistant plants can limit pathogen replication, while tolerant hosts are not damaged as severely by the pathogen (Pagán & García‐Arenal, 2018). Both traits lie on a spectrum, and, in practice, plants are more likely to have only partial (‘quantitative’) disease resistance or tolerance (French et al., 2016). Previous work has shown that tolerant and resistant crops caused contrasting outcomes for 'non‐controllers' (i.e., those that do not use an improved crop; ‘controllers’ are those that use either of the improved crop varieties; Murray‐Watson & Cunniffe, 2022). Planting a tolerant crop was often more beneficial for controllers than a resistant crop, as it limited the yield loss in infected fields. However, their reduced symptom expression additionally meant that tolerant crops could not be rogued as effectively, leading to a build‐up of inoculum and high infection levels. This lowered yields for all non‐controllers. These effects directly incentivized use of the tolerant crop, as growers could expect to earn higher profits using a tolerant crop than when they used the unimproved variety.

Conversely, as resistant crops are less susceptible to infection and limit pathogen replication once infected, the overall disease pressure on other fields is reduced when a resistant crop is used. This increases the profits of non‐controllers, as they can gain some of the benefits of resistant varieties without themselves paying the cost of the improved variety (i.e., can free ride off controllers). Therefore, the broader consequences of each crop type—the increase and decrease in infection pressure and the subsequent effect on profits of non‐controllers—can be considered externalities of each control decision. In Murray‐Watson and Cunniffe (2022), growers could not choose directly between resistant and tolerant crops, so the impact of these externalities on growers' disease management practices when growers can choose between three crop types—unimproved, tolerant or resistant—is unknown.

As a case study, we examine the effect of this three‐way choice using tomato yellow leaf curl virus (TYLCV). TYLCV is a global threat to tomato (*Solanum lycopsersicum*) production, and it has been detected in Australia, North America, Europe and East Asia (Ramos et al., 2019). The virus is transmitted by *Bemisia tabaci* (Pan et al., 2012) and infection causes leaf curling and chlorosis, stunting and up to 100% yield loss (Dhaliwal et al., 2020). Recently, tomato varieties that are either tolerant or resistant to TYLCV have been deployed (Riley & Srinivasan, 2019), helping to reduce yield loss. Other control mechanisms can be implemented as part of an integrated pest management (IPM) strategy, including the application of silver mulch, roguing or cyantraniliprole insecticide treatment (Polston et al., 1999; Riley & Srinivasan, 2019).

We consider the perspective of a social planner who has the ability to subsidize these crop types. Historically, the European Union has subsidized the cultivation of processing tomato varieties (Sumner et al., 2001); here, we study subsidies as a means of promoting particular epidemic outcomes. Subsidies are a means of rewarding/penalizing a grower based on the external effects of their actions (Pretty et al., 2001). They have a long history in agriculture, from regulating food production to promoting more environmentally friendly practices such as hedgerow conservation in the United Kingdom (Stokstad, 2020). They have also been used to encourage the use of particular crop varieties or control strategies (Okechukwu & Kumar, 2016). We considered two subsidies: one for a resistant crop and one for a tolerant crop, either of which is provided to the growers by a social planner or centralized body. As, in our model, growers choose between different crop varieties based on their expected profitability, subsidizing each crop type incentivizes its use. The planner has two objectives: to maximize the average profit across growers and to minimize their own spending on subsidies. We studied the options available to the social planner using Pareto optimality.

Thus, this work addresses three primary questions: (1) what are the long‐term outcomes when growers have access to three different crop varieties? (2) How do the responses depend on the efficacy and cost of tolerant and resistant traits? and (3) Which subsidization strategy promotes socially optimal solutions?

## 2 METHODS

### 2.1 Epidemic model with three crop varieties

The following model extends that of Murray‐Watson et al. (2022). It describes the spread of TYLCV through a population of fields, each cultivated by a single grower. The fields can be planted with one of the three crop varieties: an unimproved crop (*U*), a crop that is tolerant to TYLCV (*T*) or a crop that is resistant to TYLCV (*R*). We also track each field's infection status; as we do not model within‐field spread, fields can either be susceptible to infection (*S*), latently infected (*E*) or a source of inoculum (*I*) (i.e., disease may spread to surrounding fields, subsequently referred to as ‘infectious’; there is no time‐dependent infectivity of infectious fields). The parameters used in this model are identical to those presented in Murray‐Watson and Cunniffe (2022), with a summary of the parameter values presented in Tables 1 and 2. Variables of a crop's infection status are scaled to be a proportion of the total number of fields (i.e., *N* = *S*_*U*_ + *E*_*U*_ + *I*_*U*_ + *S*_*T*_ + *E*_*T*_ + *I*_*T*_ + *S*_*R*_ + *E*_*R*_ + *I*_*R*_ = 1).

**TABLE 1:** Parameters related to resistant and tolerant crop varieties.

Parameter	Meaning	Value if unimproved	Value if resistant	Value if tolerant
*δ*_*βb*_	Relative susceptibility to disease	1	0.5	1
*δ*_*σb*_	Relative ability to act as source of inoculum (‘infectivity’)	1	0.5	1
*δ*_*ψb*_	Relative yield	1	1	1
*δ*_*ιb*_	Relative losses due to disease	1	1	0.1
*δ*_*ϵb*_	Relative latent period of disease	1	0.5	1
*δ*_*νb*_	Relative probability of disease detection	1	1	0.1

*Note*: The distinction between the two varieties lies in how the disease is transmitted and what losses are incurred when a field is actively infected. Here, *b* is the strategy of the grower that can be use of an unimproved (*U*), disease‐resistant (*R*) or disease‐tolerant (*T*) crop.

**TABLE 2:** Summary of parameter values.

Parameters	Meaning	Value	References
1/*γ*	Length of the growing season	120 days	Holt et al. (1999)
*β*	Rate of secondary infection	0.055/N/day/field	See main text
*∆*	Time between roguing	120 days	Illustrative
*ν*	Probability of detection	1	Illustrative
*μ*_*U*_	Removal rate (unimproved)	1/60 day^−1^	Illustrative
*μ*_*R*_	Removal rate (resistant)	1/60 day^−1^	Illustrative
*μ*_*T*_	Removal rate (tolerant)	1/1140 day^−1^	Illustrative
1/*ε*	Average latent period	41 days	Ber et al. (1990), Holt et al. (1999)
*η*	Responsiveness of growers	10	Murray‐Watson et al. (2022)
*Y*	Maximum yield	1	All values scaled relative
*ι*	Loss due to infection	0.6	Riley and Srinivasan (2019)
*ϕ*_*T*_	Cost of tolerant crop	0.1	Fonsah et al. (2018)
*ϕ*_*R*_	Cost of resistant crop	0.1	Fonsah et al. (2018)
*ϕ*_*Q*_	Relative reduction in loss due to roguing	0.7, 17	Illustrative
*N*	Total number of fields	1	Scaled to 1

Irrespective of the crop type planted in the field, and unless the crop in a field is rogued (see below), the season length (1/*γ*) is 120 days (Holt et al., 1999). Replanting of any field occurs immediately after it is harvested. Susceptible fields become infected at rate *βI* day^−1^, where *I* is the proportion of ‘infectious’ fields. As a resistant crop limits pathogen replication, it also reduces the probability of infection per unit time by a factor *δ*_*βR*_ < 1. As a tolerant crop does not reduce the probability of infection, we presume that *δ*_*βT*_ = 1. If fields are infected, they enter the ‘exposed’ compartment where they remain latently infected (i.e., infected but not ‘infectious’) for an average of 1/*ϵ* days. During this time, we assume that they show no symptoms of infection and the virus cannot spread to other fields. We assume that resistant crops that become infected have a reduced rate of symptom development, increasing this latent period by a factor of *δ*_*ϵR*_ < 1 (ensuring that *δ*_*ϵR*_/*ϵ* < 1/*ϵ*). The tolerant crop does not restrict viral replication, so does not reduce the latent period.

After fully expressing symptoms and becoming a source of viral inoculum, the ultimate fate of any field is to be either harvested or rogued. Roguing involves surveying fields (‘scouting’) and removing any visibly infected plants; this has been used as a means of TYLCV management for decades (e.g., Polston et al., 1999). Tomatoes can be harvested at different levels of maturity and ripen off‐vine (Arah et al., 2015); therefore, if there is infection in a field, it may be better for growers to harvest the entire field to prevent infection spreading. This will reduce the infection‐associated yield loss by a factor *ϕQ* such that the losses experienced by a grower who has rogued an infectious field are *ϕQι*, where *ι* is the infection‐associated yield loss. We assume scouting occurs at time intervals of ∆ days and symptoms are detected with probability *ν*. Fields with symptoms that are ‘infectious’ (class *I*_*b*_) are removed as soon as they are detected. This was done to simplify model formulation and to allow us to continue to model at the field level. Altering this assumption such that within‐field spread is allowed would change the nature of the pay‐offs associated with each strategy, as well as alter the rate of disease spread.

As tolerant crops reduce symptom severity, we presume that they have a lower probability of detection (reduced by multiplication by a factor of *δ*_*νT*_ < 1) compared to an unimproved or resistant crop (where *δ*_*νR*_ = 1).

For this work, particularly for the default parameterization, we assume that resistance acts to reduce the viral load, but once resistance is overcome, the yield loss is the same as that of an unimproved crop (as the yield loss for this crop represents an ‘average’ yield loss). However, the viral titre is reduced enough that there is still a reduced probability of virus being transmitted from a resistant crop to surrounding fields. The removal rates of fields, *μ*_*b*_ with *b* ∈ {*U*, *T*, *R*}, which represent such a programme of roguing of ‘infectious’ fields, are given by Cunniffe et al. (2014):

(1) $$\mu_{U}=\frac{1}{\left(\frac{1}{\nu }-\frac{1}{2}\right)\Delta }$$

(2) $$\mu_{T}=\frac{1}{\left(\frac{1}{\delta_{\mathrm{\nu T}}\nu }-\frac{1}{2}\right)\Delta }$$

(3) $$\mu_{R}=\frac{1}{\left(\frac{1}{\delta_{\mathrm{\nu R}}\nu }-\frac{1}{2}\right)\Delta }$$

The epidemiological model is then given by

(4) $$\frac{dS_{U}}{dt}={\gamma \theta }_{U}+M_{U}-\beta S_{U}\left(I_{U}+\delta_{\mathrm{\sigma T}}I_{T}+\delta_{\mathrm{\sigma R}}I_{R}\right)-\gamma S_{U}$$

(5) $$\frac{dE_{U}}{dt}=\beta S_{U}\left(I_{U}+\delta_{\mathrm{\sigma T}}I_{T}+\delta_{\mathrm{\sigma R}}I_{R}\right)-\epsilon E_{U}-\gamma E_{U}$$

(6) $$\frac{dI_{U}}{dt}=\epsilon E_{U}-\mu_{U}I_{U}–\gamma I_{U}$$

(7) $$\frac{dS_{T}}{dt}={\gamma \theta }_{T}+M_{T}-{\delta \beta }_{T}\beta S_{T}\left(I_{U}+\delta_{\mathrm{\sigma T}}I_{T}+\delta_{\mathrm{\sigma R}}\mathrm{IR}\right)-\gamma S_{T}$$

(8) $$\frac{dE_{T}}{dt}=\delta_{\mathrm{\beta T}}\beta S_{T}\left(I_{U}+\delta_{\mathrm{\sigma T}}I_{T}+\delta_{\mathrm{\sigma R}}I_{R}\right)-\delta_{\mathrm{\epsilon T}}\epsilon E_{T}-\gamma E_{T}$$

(9) $$\frac{dI_{T}}{dt}=\delta_{\mathrm{\epsilon T}}\epsilon E_{T}-\mu_{T}I_{T}-\gamma I_{T}$$

(10) $$\frac{dS_{T}}{dt}={\gamma \theta }_{R}+M_{R}-\delta_{\mathrm{\beta R}}\beta S_{R}\left(I_{U}+\delta_{\mathrm{\sigma T}}I_{T}+\delta_{\mathrm{\sigma R}}I_{R}\right)–\gamma S_{R}\;$$

(11) $$\frac{dE_{R}}{dt}=\beta S_{R}\left(I_{U}+\delta_{\mathrm{\sigma T}}I_{T}+\delta_{\mathrm{\sigma R}}I_{R}\right)-\delta_{\mathrm{\epsilon R}}\epsilon E_{R}-\gamma E_{R}$$

(12) $$\frac{dI_{R}}{dt}=\delta_{\mathrm{\epsilon R}}\epsilon E_{R}-\mu_{R}I_{R}-\gamma I_{R}$$

where terms *γθ*_*U*_, *γθ*_*T*_ and *γθ*_*R*_ are the rates of replanting for harvested fields, while *M*_*U*_, *M*_*R*_ and *M*_*T*_ are rates of replanting for rogued fields:

(13) $$\begin{array}{c} \theta_{U}=\left(1-z_{\mathrm{SU}}\right)S_{U}+\left(1-z_{\mathrm{EU}}\right)E_{U}+\left(1-z_{\mathrm{IHU}}\right)I_{U}+z_{\mathrm{STU}}S_{T}\\ \;+z_{\mathrm{ETU}}E_{T}+z_{\text{IHTU}}I_{T}+z_{\mathrm{SRU}}S_{R}+z_{\mathrm{ERU}}E_{R}+z_{\text{IHRU}}I_{R} \end{array}$$

(14) $$\begin{array}{c} \theta_{T}=\left(1-z_{\mathrm{ST}}\right)S_{T}+\left(1-z_{\mathrm{ET}}\right)E_{T}+\left(1-z_{\mathrm{IHT}}\right)I_{T}+z_{\mathrm{SRT}}S_{R}+z_{\mathrm{ERT}}E_{R}\\ \;+z_{\text{IHRT}}I_{R}+z_{\mathrm{SUT}}S_{U}+z_{\mathrm{EUT}}E_{U}+z_{\text{IHUT}}I_{U} \end{array}$$

(15) $$\begin{array}{c} \theta_{R}=\left(1-z_{\mathrm{SR}}\right)S_{R}+\left(1-z_{\mathrm{ER}}\right)E_{R}+\left(1-z_{\mathrm{IHR}}\right)I_{R}+z_{\mathrm{STR}}S_{T}+z_{\mathrm{ETR}}E_{T}\\ \;+z_{\text{IHTR}}I_{T}+z_{\mathrm{SUR}}S_{U}+z_{\mathrm{EUR}}E_{U}+z_{\text{IHUR}}I_{U} \end{array}$$

(16) $$M_{U}=\left(1-z_{\mathrm{IRU}}\right)\mu_{U}I_{U}+z_{\text{IRTU}}\mu_{T}I_{T}+z_{\text{IRRU}}\mu_{R}I_{R}$$

(17) $$M_{T}=z_{\text{IRUT}}\mu_{U}I_{U}+\left(1-z_{\mathrm{IRT}}\right)\mu_{T}I_{T}+z_{\text{IRRT}}\mu_{R}I_{R}$$

(18) $$M_{R}=z_{\text{IRUR}}\mu_{U}I_{U}+z_{\text{IRTR}}\mu_{T}I_{T}+\left(1-z_{\mathrm{IRR}}\right)\mu_{R}I_{R}$$

Note, a distinction is made between the replanting of rogued (*I*_*RU*_, *I*_*RT*_ or *I*_*RR*_) and harvested (*I*_*HU*_, *I*_*HT*_ or *I*_*HR*_) crops. For rogued unimproved fields, *z*_*IRU*_ *μ*_*U*_ *I*_*U*_ = *z*_*IRUT*_ *μ*_*U*_ *I*_*U*_ + *z*_*IRUR*_ *μ*_*U*_ *I*_*U*_ (i.e., the total rate at which rogued unimproved fields switch strategy must be equal to the rate at which they enter either other strategy, with equivalent expressions for tolerant and resistant crops). Similarly, for harvested unimproved fields, *z*_*IHU*_ *I*_*U*_ = *z*_*IHUT*_ *I*_*U*_ + *z*_*IHUR*_ *I*_*U*_.

**FIGURE 1:**
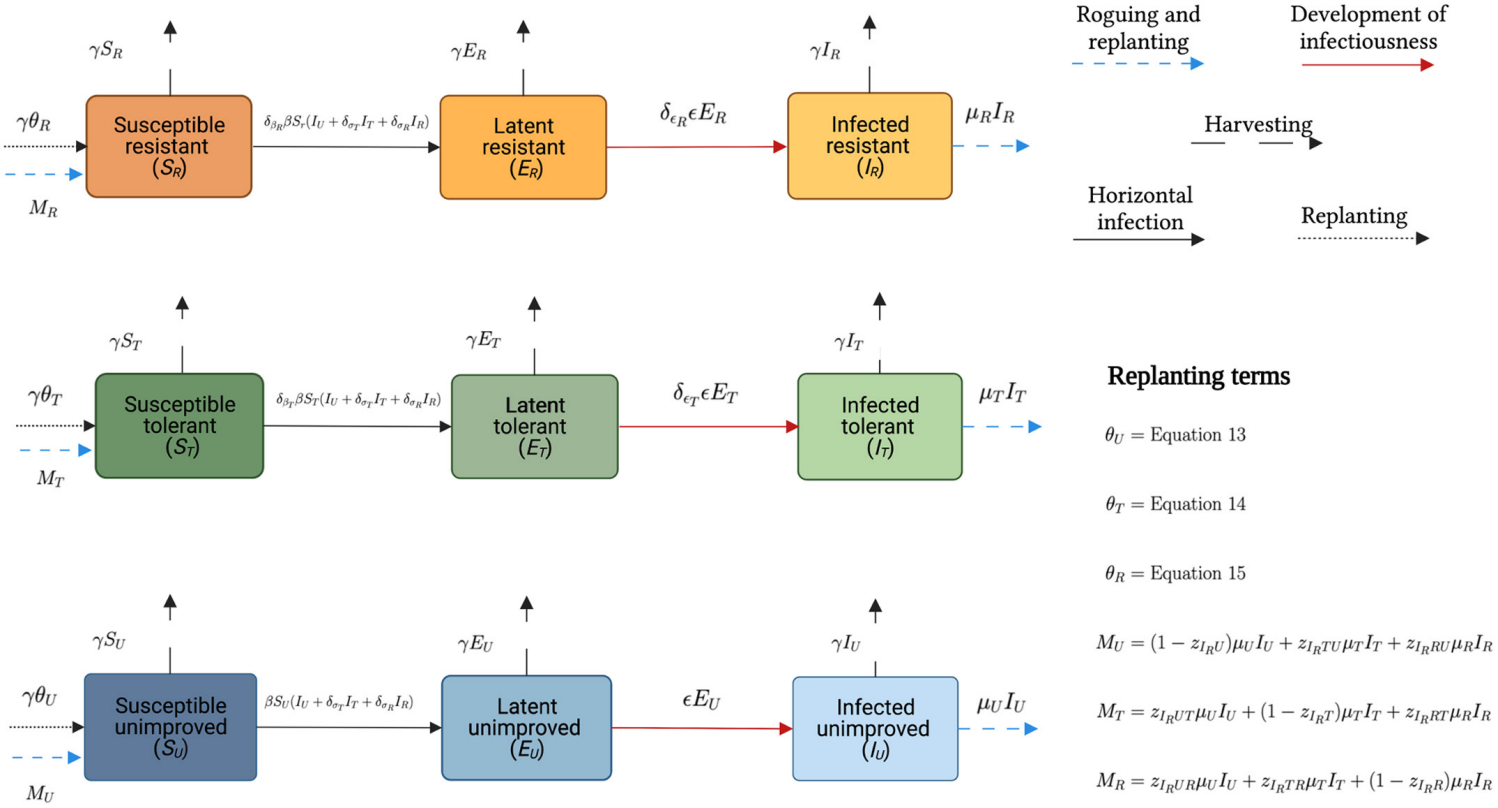
Schematic showing the structure of the model where tomato growers can choose their strategy based on expected profits. We have three classes of growers: those who use unimproved seed (subscript *U*), those who use seed tolerant to tomato yellow leaf curl virus (TYLCV; subscript *T*) and those that use seed resistant to TYLCV (subscript *R*). The terms *θ*_*U*_, *θ*_*T*_ and *θ*_*R*_ are the rates of replanting for harvested fields, while *M*_*U*_, *M*_*R*_ and *M*_*T*_ are rates of replanting for rogued fields (Equations 13–18, with *M*_*U*_ + *M*_*R*_ + *M*_*T*_ = *μ*_*U*_ *I*_*U*_ + *μ*_*R*_ *I*_*R*_ + *μ*_*T*_ *I*_*T*_). Created with BioRender.com. [Colour figure can be viewed at wileyonlinelibrary.com]

Replanting in our model is represented by switching terms (McQuaid et al., 2017; Milne et al., 2016; Murray‐Watson et al., 2022), which are the terms of the form *z*_*ab*_ or *z*_*abc*_ where *a* ∈ {*S*, *E*, *I*_*H*_, *I*_*R*_} and *b*, *c* ∈ {*U*, *T*, *R*} (note the additional complexity of our model means there is a change of notation compared to Murray‐Watson et al., 2022). The switching terms with three subscripts, *z*_*abc*_, represent the probability of a grower changing strategy from their current strategy *b* to strategy *c* based on their outcome, *a*, in the previous season (note that *b* ≠ *c*). For growers whose fields were susceptible (*S*) or exposed (with latent infection; *E*), the outcome depends solely on the epidemiological class of their field at the end of the season; however, for growers whose fields were ‘infectious’, the outcome also depends on whether that infected field was rogued (subscript *R*) or harvested (subscript *H*). The two‐subscript version, *z*_*ab*_, is the probability that a grower who harvested field type *ab* switches strategy. These terms are explained below. A schematic of the model is shown in Figure 1.

As we are interested in equilibrium values, which will be reached after a different number of seasons depending on the parameter values, we examine a range of timescales throughout the article. Each timescale is chosen to ensure that, for a given parameterization, equilibrium will be reached.

### 2.2 Growers' profits

The profit a grower earns is based on the control strategy that they previously used and their infection status at the time of harvest. Each crop type (*b* ∈ {*U*, *T*, *R*}) has specific associated costs and yield losses when infected with TYLCV. We note that, in the absence of disease, we assume that all varieties have the same maximum yield, *Y*. The outcomes for each profit are based on both the control strategy used by the grower, their disease status at the time of harvest, and whether or not they rogued an *I* (with symptoms and ‘infectious’) field. The use of either the resistant or tolerant crop has an associated cost, *ϕ*_*b*_. An *I* field will incur a yield loss, *ι*, the value of which will depend on whether the field was planted with a tolerant crop or whether it was rogued before harvest. Roguing preserves some of the yield that would have been lost had the field remained undetected and was harvested later.

(19) $$\begin{array}{c} P_{\mathrm{Sb}}=\text{Profit for}\;a\;\text{susceptible field of type}\;b=\psi -\phi_{b} \end{array}$$

(20) $$\begin{array}{c} P_{\mathrm{Eb}}=\text{Profit for}\;a\;\text{latently infected field of type}\;b=\psi -\phi_{b} \end{array}$$

(21) $$\begin{array}{c} P_{\mathrm{IHb}}=\text{Profit for an infected field that was not rogued of type}\;\\ \;b=\psi -\phi_{b}-\delta_{\mathrm{\iota b}}\iota \end{array}$$

(22) $$\begin{array}{c} P_{\mathrm{IRb}}=\text{Profit for an infected field that was rogued of type}\;\\ \;b=\psi -\phi_{b}-\phi_{Q}\delta_{\mathrm{\iota b}}\iota \end{array}$$

Notably, here, *ϕ*_*U*_ = 0, because *ϕ*_*b*_ represents the additional cost of using crop type *b*. For a full enumeration of the outcomes for growers, see Appendix S1.

For our parameterization, we assume that latently infected fields do not lose any yield, so *P*_*Sb*_ = *P*_*Eb*_, *b* ∈ {*U*, *T*, *R*}. As growers who use an unimproved crop and harvest susceptible or latently infected fields pay no cost of control and sustain no yield loss, *P*_*SU*_ = *P*_*EU*_ is always the maximum achievable profit. The relative sizes of the remaining profits will depend on the values of the costs of the improved varieties (*ϕ*_*T*_ and *ϕ*_*R*_), the infection‐induced yield losses (*δ*_*ιU*_ *ι*, *δ*_*ιT*_ *ι* and *δ*_*ιR*_ *ι*) and the benefit of roguing (*ϕ*_*Q*_). Under the default parameterization (Table 2), the ordering of the profits is given by

(23) $$\begin{array}{c} P_{\mathrm{SU}}=P_{\mathrm{EU}}>P_{\mathrm{ST}}=P_{\mathrm{ET}}=P_{\mathrm{SR}}=P_{\mathrm{ER}}>P_{\mathrm{IRT}}>P_{\mathrm{IHT}}>P_{\mathrm{IRU}}>P_{\mathrm{IHU}}\\ \;>P_{\mathrm{IRR}}>P_{\mathrm{IHR}} \end{array}$$

### 2.3 Calculating expected profits

The growers' decision of which crop variety to use will depend on how each individual grower's profit from the previous season compares to the expected profit of the alternative crop types. These expected profits are based on the probability of attaining each outcome outlined in Equations 42–53 (Appendix S1), which, in turn, depends on the probability of infection for each crop type. The complete derivation is provided in Appendix S1; after routine algebraic manipulations, the simplified expressions for the expected profits for unimproved (*P*_*U*_), tolerant (*P*_*T*_) and resistant (*P*_*R*_) are:

(24) $$P_{U}=\psi -q_{U}\frac{\epsilon }{\varepsilon +\gamma }\iota \left(\frac{\gamma }{\gamma +\mu_{U}}+\frac{\mu_{U}}{\mu_{U}+\gamma }\phi_{Q}\right)$$

(25) $$P_{T}=\delta_{\mathrm{\psi T}}\psi -\phi_{T}-q_{T}\frac{\delta_{\mathrm{\epsilon T}}\epsilon }{\delta_{\mathrm{\epsilon T}}\epsilon +\gamma }\delta_{\mathrm{\iota T}}L\left(\frac{\gamma }{\gamma +\mu_{T}}+\frac{\mu_{T}}{\mu_{T}+\gamma }\phi_{Q}\right)$$

(26) $$P_{R}=\delta_{\mathrm{\psi R}}\psi -\phi_{R}-q_{R}\frac{\delta_{\mathrm{\epsilon R}}\epsilon }{\delta_{\mathrm{\epsilon R}}+\gamma }\delta_{\mathrm{\iota R}}\iota \left(\frac{\gamma }{\gamma +\mu_{R}}+\frac{\mu_{R}}{\mu_{R}+\gamma }\phi_{Q}\right)$$

The purpose of the expected profit calculations is to use the current state of the system (in terms of the probability of infection) to estimate what a grower could expect to earn over the following season if those probabilities of infection remained constant. They form part of the ‘strategic‐adaptive’ expectations that growers will use—alongside their own profits from the previous season—to judge whether they should switch strategy.

### 2.4 Switching terms

The switching terms (Murray‐Watson et al., 2022) affect the rate at which growers move from their current strategy to one of the alternative strategies.

In previous models (McQuaid et al., 2017; Milne et al., 2016; Murray‐Watson et al., 2022; Saikai et al., 2021), growers only had one alternative strategy with which to compare profits. Now, growers must consider two alternatives to their current strategy. Growers first assess the expected profits of the two alternative strategies and only compare their outcome with the highest expected profit of the two alternatives. If both alternatives have the same expected profit (so, e.g., if the expected pay‐off for tolerance and resistance are the same; *P*_*T*_ = *P*_*R*_), and therefore the grower should have the same probability of switching into each strategy, growers chose to compare with the profit associated with the crop type most growers use. This follows from ‘descriptive norms’, where individuals follow what the majority of other people are doing (Cialdini et al., 1990). Additionally, there is some evidence that growers will be more likely to participate in control if other growers also participate (Milne et al., 2018). If both the expected profits and the proportion of growers using each strategy are the same, then half of the growers changing strategy will go to each alternative.

In their general form, the switching terms have the following structure, where a grower with outcome *P*_*ab*_, contemplates switching into strategy *c* or *d*, *a* ∈ {*S*, *E*, *I*_*H*_, *I*_*R*_} and *b*, *c*, *i* ∈ {*U*, *T*, *R*}, *b* ≠ *c* ≠ *d*.

If *P*_*c*_ > *P*_*d*_ or *P*_*c*_ = *P*_*d*_ and *C* > *D* (where *C* and *D* are the proportion using strategies *c* and *d*), then

(27) $$z_{\mathrm{abc}}=\max\left(0,1-\exp\left(-\eta \left(P_{c}-P_{\mathrm{ab}}\right)\right)\right)$$

(28) $$z_{\mathrm{abd}}=0$$

In the case where both the profits and the proportion of growers using the alternative strategy are equal, growers changing strategy will be divided evenly between the two alternative strategies. So, if *P*_*c*_ = *P*_*d*_ and *C* = *D*, then

(29) $$z_{\mathrm{abc}}=\frac{\left[\max\left(0,1-\exp\left(-\eta \left(P_{c}-P_{\mathrm{ab}}\right)\right)\right)\right]}{2},$$

(30) $$z_{\mathrm{abd}}=\frac{\left[\max\left(0,1-\exp\left(-\eta \left(P_{c}-P_{\mathrm{ab}}\right)\right)\right)\right]}{2},$$

To simplify writing the model, we can then say

(31) $$z_{\mathrm{ab}}=\max\left(z_{\mathrm{abc}},z_{\mathrm{abd}}\right)$$

where *z*_*ab*_ is the probability that a grower who harvested a field of type *ab* leaves strategy *b* to adopt either strategy *c* or *d*. Full details of the switching terms are given in Appendix S2.

### 2.5 Calculating the Pareto front and Gini coefficients

We use the concept of Pareto efficiency to dually optimize two conflicting objectives. Our first objective is to maximize the average profit of growers at equilibrium, calculated as the yield they achieve less any investment they have made to control for disease (based on the outcomes outlined in Equations (19), (20), (21), (22)). Our second objective is to minimize the spending of a central planning body that subsidizes the cost of tolerant and resistant crops.

If the cost to the planner of the crop type is *ϕ*_*T*,max_, and the cost paid by growers is *ϕ*_*T*_, then the subsidy to growers for the use of a tolerant crop *σ*_*T*_ = *ϕ*_*T*,max_ *− ϕ*_*T*_ (and, equivalently, for resistant crop is *σ*_*R*_ = *ϕ*_*R*,max_ − *ϕ*_*R*_). The total cost to the planner is then

(32) $$\tau =\sigma_{T}T+\sigma_{R}R$$

where *T* = *S*_*T*_ + *E*_*T*_ + *I*_*T*_ and *R* = *S*_*R*_ + *E*_*R*_ + *I*_*R*_ (i.e., are the proportions of growers using tolerant and resistant crops, respectively). We use Pareto optimality to establish the optimal allocation of subsidies between tolerant and resistant crops.

We first run the model for different values of *ϕ*_*T*_ and *ϕ*_*R*_ (400 values between 0 and 0.4 for each parameter) to find the equilibrium profit of growers and cost to the planner. We then use these two results as inputs to *get frontier*() function from the KraljicMatrix package in R (Boehmke et al., 2017), which calculates the Pareto front.

Though the Pareto front shows the optimal outcomes for a given set of parameters, one objective may be more heavily prioritized over the other. To quantify the degree of fairness between outcomes, we calculate the Gini coefficient (Gini, 1936). Originally developed as a measure of income inequality, the Gini coefficient calculates the extent to which a solution deviates from total equality between objectives. In that sense, the Gini coefficient is related to fairness but does not consider the optimality of the solution: a scenario with a low Gini coefficient (indicating a high degree of fairness between objectives, with a value of zero indicating perfect equality) may be a suboptimal solution for one or both objectives. Similarly, in some scenarios, the Gini coefficient may be small due to the number of objectives being considered: if just one individual's objective is perfectly optimized, and all others are ignored, then *G* = 1 – 1/*n*, where *n* is the number of objectives. The coefficient has been used to estimate regional inequalities in the risk of veterinary epidemics (Li et al., 2020), geographical variability in incidence of sexually transmitted diseases (Elliott et al., 2002) and in ecology to evaluate management programmes and agricultural productivity (Zaffaroni & Bevacqua, 2022). The Gini coefficient for a given scenario *i* is calculated by Dorfman (1979) and Zaffaroni and Bevacqua (2022):

(33) $$G_{i}=\frac{\sum_{j=1}^{n}\sum_{z=1}^{n}\left|x_{j,i}-x_{z,i}\right|}{2n\sum_{j=1}^{n}x_{j,i}}$$

where *n* is the number of objectives considered and *x*_*j*,*i*_ and *x*_*z*,*i*_ are the values of the objectives for scenario *i*.

Calculating the Gini coefficient requires both objectives to be measured on the same scale, so we must aim to maximize or minimize both objectives. We therefore calculate the relative cost to the planner, τ* as

(34) $$\tau^{*}=\frac{\max\left(\tau \right)-\tau_{j}}{\max\left(\tau \right)-\min\left(\tau \right)}$$

where max(*τ*) and min(*τ*) are the maximum and minimum costs to the planner that lie along the Pareto front and *τ*_*j*_ is the cost value for a combination of *ϕ*_*R*_ and *ϕ*_*T*_. This scales the cost to the planner between 0 and 1. Maximizing this relative cost, τ*, is equivalent to minimizing the actual cost, *τ*.

Similarly, we must also rescale the profits so that they are normalized between 0 and 1:

(35) $$P^{*}=\frac{\max\left(\text{Profit}\right)-{\text{Profit}}_{j}}{\max\left(\text{Profit}\right)-\min\left(\text{Profit}\right)}$$

where max(Profit) and min(Profit) are the maximum and minimum profits to growers that lie along the Pareto front, Profit_*j*_ is the objective value for a particular combination of *ϕ*_*R*_ and *ϕ*_*T*_.

We use the Gini coefficient to evaluate how a particular subsidy scheme benefits the planner relative to the growers, giving a measure of the fairness of each scheme. Because we have only two objectives, the Gini coefficient will be between 0 and 0.5.

## 3 RESULTS

### 3.1 Equilibria for the three‐strategy model

Using the next‐generation method (NGM; van den Driessche, 2017; Appendix S3), we find the basic reproduction number, R_0_, for the model to be

(36) $$R_{0}=\frac{\beta_{\epsilon }N}{\left(\epsilon +\gamma \right)\left(\mu_{U}+\gamma \right)}$$

At the disease‐free equilibrium, only the unimproved crop is used by growers. The absence of disease makes control obsolete, so no growers should pay the cost of control. In Equation 36, $\frac{\epsilon }{\left(\epsilon +\gamma \right)}$ is the probability that an unimproved field became ‘infectious’ before it is harvested and $\frac{1}{\left(\mu_{U}+\gamma \right)}$ is the mean time fields remained in the *I* compartment before they are either removed via roguing or harvested. The number of infections caused by these infectious fields is *βN*. There are eight possible long‐term outcomes for the model:

- Disease‐free equilibrium (DFE): where *R*_0_ < 1 and no growers control for disease.
- ‘No control’ equilibrium: where disease is endemic but no growers use an improved crop (*N* = *U*).
- ‘All tolerant’ equilibrium: where disease is endemic and growers only use a tolerant crop (*N* = *T*).
- ‘All resistant’ equilibrium: where disease is endemic and growers only use a resistant crop (*N* = *R*).
- Three‐strategy equilibrium: where disease is endemic and crops of all three varieties are used (*N* = *U* + *T* + *R*).
- ‘Tolerant and unimproved’ equilibrium: where disease is endemic and growers use either a tolerant or unimproved crop (*N* = *T* + *U*).
- ‘Resistant and unimproved’ equilibrium: where disease is endemic and growers use either a resistant or unimproved crop (*N* = *R* + *U*).
- ‘Tolerant and resistant’ equilibrium: where disease is endemic and growers use either a tolerant or resistant crop (*N* = *T* + *R*).

Notably, the growers in the ‘no control’ equilibrium still rogue their fields; throughout this article, we use ‘no control’ as a byword for those that do not use any improved crop variety.

Additionally, the model presented in Murray‐Watson and Cunniffe (2022) allowed for a bistable region when *R*_0_ < 1. In this bistable region, the system could equilibrate at either the disease‐free equilibrium or the all‐tolerant equilibrium, depending on the initial conditions. Murray‐Watson and Cunniffe (2022) found that the all‐tolerant equilibrium was more likely where the initial proportion of those using a tolerant crop (*S*_*T*_(0) + *E*_*T*_(0) + *I*_*T*_(0)), and the initial proportion of ‘infectious’ tolerant fields (*I*_*T*_(0)), was high. To prevent this from affecting our results, we always begin the models with the initial conditions outlined in Table 3, which guarantees a disease‐free equilibrium in the bistable region for the default parameterization.

**TABLE 3:** Default initial conditions, terms for growers harvesting or roguing fields with active infections and notation for switching terms.

Variable	Meaning	Value
*S* *_U_* (0)	Initial proportion of susceptible unimproved fields	0.79
*E*_*U*_ (0)	Initial proportion of latently infected unimproved fields	0
*I*_*U*_ (0)	Initial proportion of infectious unimproved fields	0.01
*S*_*T*_ (0)	Initial proportion of susceptible tolerant fields	0.1
*E*_*T*_ (0)	Initial proportion of latently infected tolerant fields	0
*I*_*T*_ (0)	Initial proportion of actively infected tolerant fields	0
*S*_*R*_ (0)	Initial proportion of susceptible resistant fields	0.1
*E*_*R*_ (0)	Initial proportion of latently infected resistant fields	0
*I*_*R*_ (0)	Initial proportion of actively infected resistant fields	0
*I*_*HU*_	Actively infected, harvested, unimproved field	–
*I*_*RU*_	Actively infected, rogued, unimproved field	–
*I*_*HT*_	Actively infected, harvested, tolerant field	–
*I*_*RT*_	Actively infected, rogued, tolerant field	–
*I*_*HR*_	Actively infected, harvested, resistant field	–
*I*_*RR*_	Actively infected, rogued, resistant field	–
*C*	Proportion using strategy *c* (*c ∈ U*, *T*, *R*)	–
*D*	Proportion using strategy *d* (*d ∈ U*, *T*, *R*)	–

*Note*: The initial conditions presented here led to a disease‐free equilibrium.

**FIGURE 2:**
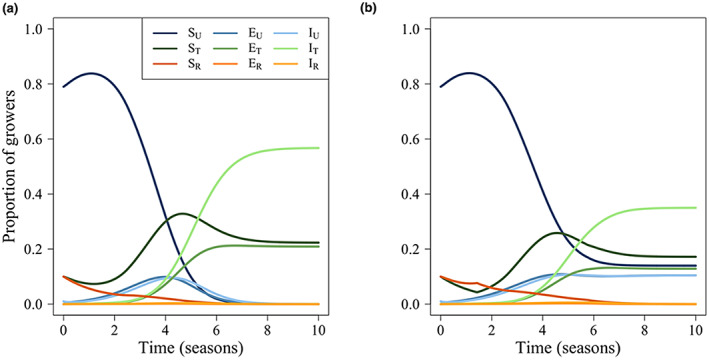
Model dynamics for a case study showing the proportion of tomato growers using crops that are unimproved (subscript *U*), tolerant (subscript *T*) or resistant (subscript *R*) to tomato yellow leaf curl virus (TYLCV). Fields are either susceptible (*S*), latently infected (*E*) or actively infected and a source of viral inoculum (*I*). (a) Dynamics resulting from the default parameterization presented in Table 1. (b) Dynamics when the tolerant crop is less effective (i.e., *δ*_*ιT*_ = 0.5). All initial conditions are as presented in Table 3. [Colour figure can be viewed at wileyonlinelibrary.com]

Default dynamics for the model are presented in Figure 2a; dynamics for a less effective tolerant crop (where *δ*_*ιT*_ = 0.5) are in Figure 2b. Under the default parameterization, there is an ‘all tolerant’ equilibrium; reducing the effectiveness of the tolerant crop gives an ‘unimproved and tolerant’ equilibrium. The transient dynamics show that initially, when disease prevalence is low, the unimproved crop is most beneficial. However, as disease prevalence increases, use of the tolerant crop also increases.

**FIGURE 3:**
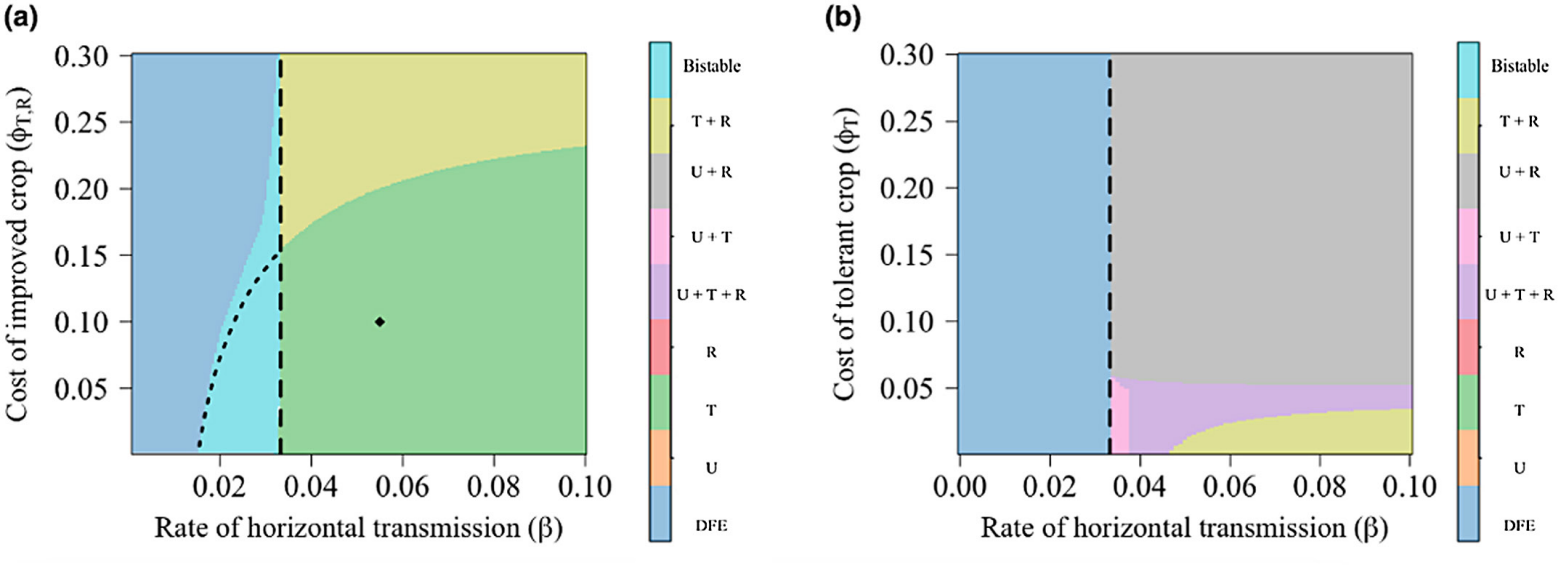
Effect of the cost of tolerant or resistant crop and the rate of horizontal disease transmission on the equilibrium values. In (a) the default parameterization are used (Tables 1 and 2), while (b) has the cost of resistant crop(*ϕ*_*R*_) = 0.06, and the relative probability of disease detection in the tolerant crop(*δ*_*νT*_) = 1 (as might be the case if disease detection was done via a genetic test), relative losses due to disease (*δ*_*ιT*_) = 0.3 and relative susceptibility to disease of resistant crop (*δ*_*βR*_) = 0.1. In both cases, a disease‐free equilibrium (DFE) persists once the basic reproduction number for the disease (R_0_) < 1, though there is a bistable region shown in light blue between the limits of *β*_*U*_ = 0.020 and 0.0333 day^−1^ in (a). The equilibrium realized within this region is either a mixed tolerant (T) and unimproved (U) crop equilibrium or an all‐tolerant equilibrium (indicated by the dotted line). In (b), lowering the costs and losses associated with the resistant crop allowed for a mixed equilibrium between resistant (R) and unimproved crop and a three‐strategy mixed equilibrium between all three crop types. The dashed vertical lines show R_0_ = 1. [Colour figure can be viewed at wileyonlinelibrary.com]

Parameterizations that lead to the disease‐free, all‐tolerant, tolerant and resistant, unimproved and resistant, unimproved and tolerant, and three‐strategy equilibria are shown in Figure 3, in which we vary the rate of horizontal transmission (*β*) and, in (a), the cost of both improved crops (*ϕ*_*R*_ and *ϕ*_*T*_) and, in (b), just *ϕ*_*T*_ (*ϕ*_*R*_ is fixed at 0.06 for demonstrative purposes). However, the externalities generated by both tolerant and resistant crops mean that some of these potential equilibria were only realized within a narrow range of parameter values (namely the ‘no control’ equilibrium). The ‘all resistant’ equilibrium is not possible, as those using an unimproved crop can free ride off the efforts of those who plant a resistant crop (Murray‐Watson & Cunniffe, 2022).

Which long‐term outcomes were possible depends strongly on parameter values, particularly the parameterization of resistance and tolerance. When the cost of both improved crop varieties varied (Figure 3a), lower costs incentivized the use of a tolerant crop, and as the rate of horizontal transmission (*β*) increased, more growers used the tolerant crop. The high probability of infection meant that growers would be better off using a tolerant crop and limiting the damage due to disease (as, under this parameterization, the losses in the tolerant crop were a tenth of those for the resistant or unimproved crop). As the benefits of the tolerant crop are felt privately by those who use them, there is more incentive to use these varieties and an ‘all‐tolerant’ equilibrium is possible even at relatively high costs of control. A fuller exploration of how equilibria are affected by parameter values relating to tolerance and resistance is provided in Appendix S4.

If we keep the cost of the resistant crop fixed at a low price (*ϕ*_*R*_ = 0.06) and parameterize it such that it is significantly less susceptible to infection (*δ*_*βR*_ = 0.1), its use is much more widespread (Figure 3b). At low values of *ϕ*_*T*_, mixed equilibria with the tolerant crop were achieved, particularly at higher values of *β* when infection becomes more likely to occur. However, under this parameterization, the tolerant crop is both more easily detected via roguing (*δ*_*νR*_ = 1) and is less effective at limiting yield loss (*δ*_*ιT*_ = 0.3), so there is less of an incentive for growers to use it compared to the parameterization in Figure 3a. As *ϕ*_*T*_ increases, growers stop using the tolerant crop and, instead, a larger proportion free ride off of the efforts of those that use the resistant crop. As the resistant crop generates positive externalities for other growers, the fields of the non‐controllers have a reduced infection pressure without incurring any costs. Consequently, an ‘all‐resistant’ equilibrium is not reached.

The bistable region between *β* = 0.02 and 0.0333 day^−1^ in Figure 3a varies between either the DFE and ‘all tolerant’ equilibria or the DFE and ‘tolerant and resistant’ equilibria depending on parameter values, with the dotted line showing the distinction between these equilibria. As in Murray‐Watson and Cunniffe (2022), a high initial proportion of tolerant crops (or ‘infectious’ crops) causes the system to go to the disease‐endemic equilibria.

### 3.2 Effect of parameters relating to tolerance and resistance on behaviour

**FIGURE 4:**
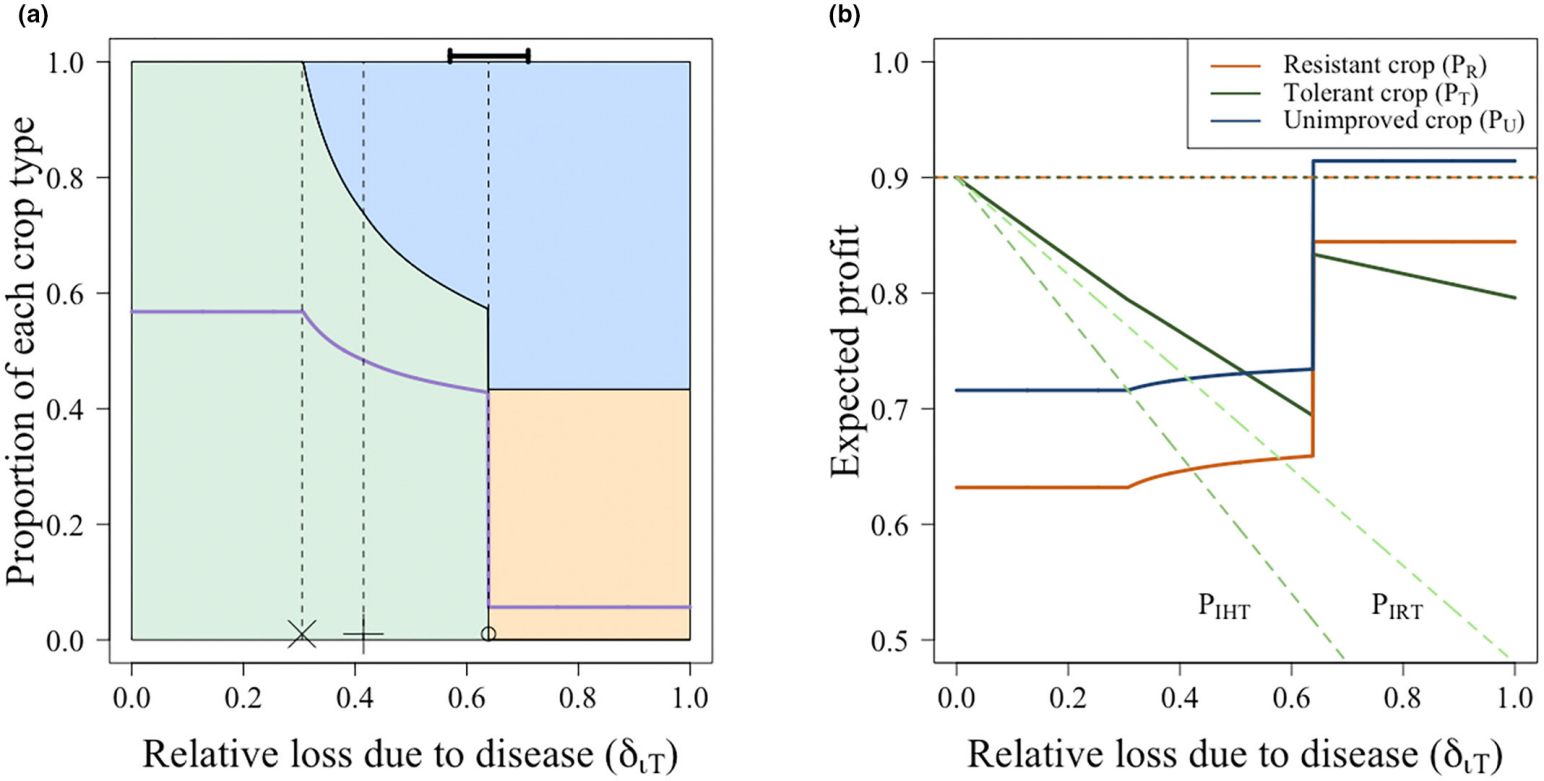
Effect of the relative loss due to infection on the choice of unimproved, tolerant or resistant crop and on the profits of each strategy at equilibrium. (a) Effect of relative loss on the proportion of actively infected (*I*_*T*_ + *I*_*R*_ + *I*_*U*_), unimproved (*U*), tolerant (*T*) and resistant (*R*) crops chosen. (b) The expected profit for growers using unimproved, resistant or tolerant crop with increasing susceptibility. The dashed orange lines show the profit for susceptible and latently infected tolerant and resistant fields (*P*_*ST*_ = *P*_*ET*_ = *P*_*SR*_ = *P*_*ER*_ = 0.9), and the green dashed lines show the profits for a rogued and harvested infected tolerant field. For low values of *δ*_*ιT*_, all growers use the tolerant crop. Once *δ*_*ιT*_ > 0.64 (when *P*_*T*_ = *P*_*R*_), all non‐controllers (using the unimproved crop) who harvested an infected crop should consider switching to the resistant crop, rather than the tolerant crop. This causes a decrease in those using the tolerant crop and an increase in those planting the resistant crop, which also causes a decrease in the proportion of infected fields (a). The black bar in (a) shows the region where bistability affects the switch between equilibria. Save for *δ*_*ιT*_, parameters and initial conditions are as in Tables 2 and 3, respectively. [Colour figure can be viewed at wileyonlinelibrary.com]

The primary characteristic of a tolerant crop is its ability to limit yield loss if infection occurs, so the losses experienced by growers of the tolerant crop were lower than those of the unimproved or resistant crop (*δ*_*ιT*_ *ι* < *ι*). Therefore, at low values of *δ*_*ιT*_ (Figure 4), all growers use the tolerant crop. As the tolerant crop is less likely to be rogued, infection builds up, further incentivizing growers to use the tolerant crop.

As *δ*_*ιT*_ increases, the value of the expected profit of those growing the tolerant crop (*P*_*T*_) falls. For this parameterization, at *δ*_*ιT*_ ≈ 0.31 (‘X’), the profits of growers who harvested infected tolerant crops were lower than the expected profits of non‐controllers (*P*_IHT_ < *P*_*U*_), so controllers that have harvested an infected crop have a non‐zero probability of switching strategy. As the expected profit for the unimproved crop is higher than that of the resistant crop (*P*_*U*_ > *P*_*R*_), these growers only consider switching to the unimproved crop. Similarly, at *δ*_*ιT*_ ≈ 0.405 (‘+’), *P*_IRT_ < *P*_*U*_ and growers of the tolerant crop who have rogued their fields should also consider switching to the unimproved crop. These changes to the switching terms cause sharp changes in the proportion of growers using each strategy.

In Figure 4, at the value of *δ*_*ιT*_ ≈ 0.64 (‘O’), growers of the tolerant crop who have rogued their infected fields earn less than the expected profit of those using the resistant crop (*P*_IRT_ < *P*_*R*_). These growers start switching strategy, causing a decrease in the proportion using tolerant crop and an increase in the expected profits of both the tolerant and the resistant crops. At *δ*_*ιT*_ ≈ 0.64 *P*_*T*_ = *P*_*R*_: the model set‐up means that growers of an unimproved crop, who harvested infected fields, compare with the profit of whichever strategy has more growers using it, which, in this case, is the tolerant crop. Once *δ*_*ιT*_ > 0.64 (‘O’), *P*_*T*_ < *P*_*R*_ and the growers of an infected unimproved crop now consider switching to the resistant crop. This causes a sharp increase in those using the resistant crop and decrease in proportion of infected fields (Figure 4a at *δ*_*ιT*_ > 0.64). All expected profits rise with this decrease in infection (Figure 4b), though the relative ordering of the profits does not change (*P*_*U*_ > *P*_*R*_ > *P*_*T*_). The response flattens out after *δ*_*ιT*_ > 0.64 as no growers were using the tolerant crop, so no growers were affected by the changes in this parameter. Therefore, the model ran to the same equilibrium irrespective of further changes to *δ*_*ιT*_.

Between the values of *δ*_*ιT*_ ≈ 0.51 and 0.71 (denoted by the black bar in Figure 4a), there is bistability, which changes the precise value of *δ*_*ιT*_ at which growers stop using the tolerant crop (‘O’), as a function of the initial conditions of the model (Appendix S3).

**FIGURE 5:**
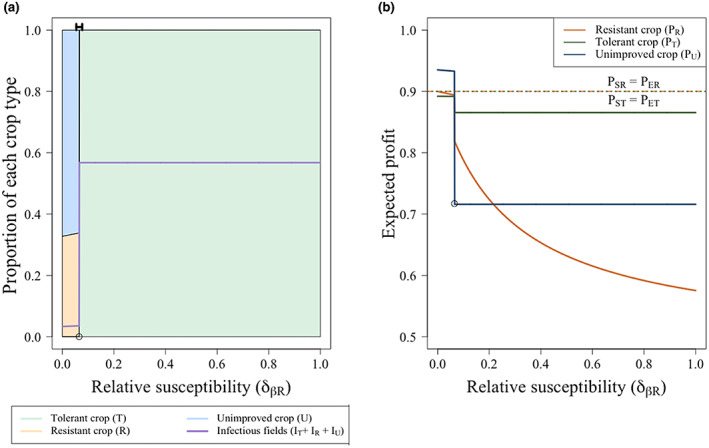
Effect of the relative susceptibility to infection (*δ*_*βR*_) of a crop on the choice of unimproved, tolerant or resistant crop. (a) Effect of increasing *δ*_*βR*_ on the proportion of actively infected (*I*_*T*_ + *I*_*R*_ + *I*_*U*_), unimproved (*U*), tolerant (*T*) and resistant (*R*) fields grown. (b) The expected profit for growers using unimproved, resistant or tolerant crop, with increasing *δ*_*βR*_. The dashed orange lines show the profit for susceptible and latently infected tolerant and resistant fields (*P*_*ST*_ = *P*_*ET*_ = *P*_*SR*_ = *P*_*ER*_ = 0.9). At low values of *δ*_*βR*_, most growers use the resistant crop. However, as *δ*_*βR*_ increases, the benefits to growers of the resistant crop are lower, and growers start using the tolerant crop. This causes an increase in the proportion of infected fields. Save for *δ*_*βR*_, parameters and initial conditions are as in Tables 2 and 3 respectively. [Colour figure can be viewed at wileyonlinelibrary.com]

The resistant crop only exists at equilibrium when the relative susceptibility of resistant plants (*δ*_*βR*_) is low (Figure 5). Once the resistant crop increases in susceptibility to infection, growers prefer to use the tolerant crop and sustain lower losses once infection occurs. For the parameterization in Table 2, and as *δ*_*βR*_ varies, when *δ*_*βR*_ = 0.08, all growers use the tolerant crop (‘all tolerant’ equilibrium); therefore, the responses to changes in *δ*_*βR*_ flatten out as there are no resistant fields with reduced susceptibility. The remaining parameters are unchanged, so the model goes to the same equilibrium as parameter *δ*_*βR*_ is varied.

Similarly to Figure 4, between the values of *δ*_*βR*_ = 0.054 and 0.077 (denoted by the black bar in Figure 5), there is bistability that changes the point at which growers stop using the resistant crop and only use the tolerant crop (Appendix S3).

### 3.3 Pareto optimality

**FIGURE 6:**
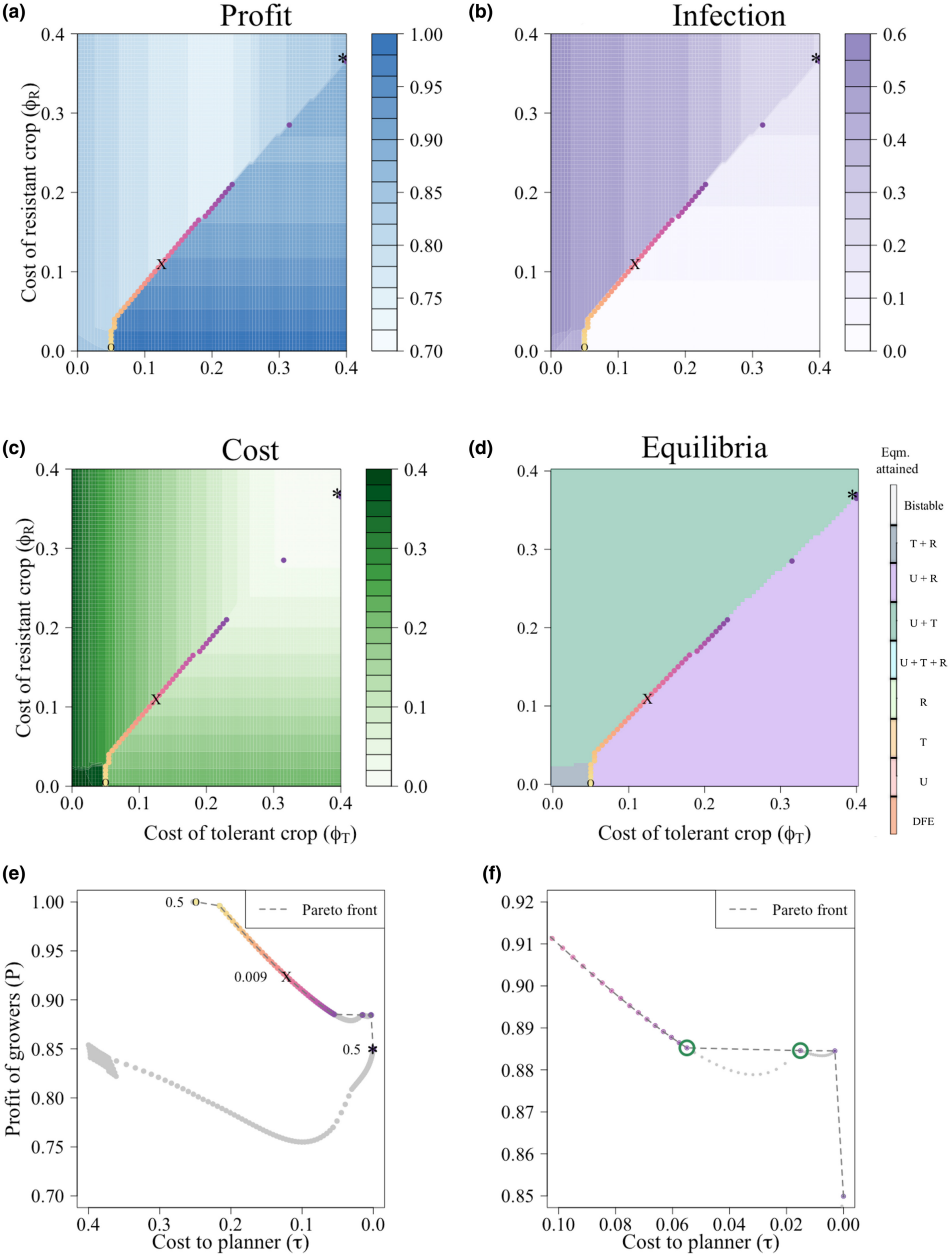
Determination of a set of solutions with equally good outcomes (Pareto front) for minimizing the cost of a subsidy scheme while maximizing the average profit of growers when both the tolerant and the resistant crop are only moderately effective. (a) The profit to growers, represented by shades of blue (Profit = *UP*_*U*_ + *TP*_*T*_ + *RP*_*R*_); (b) the proportion of actively infected fields, represented by shades of purple (Infectious = *I*_*U*_ + *I*_*T*_ + *I*_*R*_); (c) the cost to the planner of the subsidy scheme (*τ* = *σ*_*T*_ *T* + *σ*_*R*_ *R*, Equation 32); and (d) the equilibria attained (optimal outcome for both grower and planner), with increasing costs of tolerant and resistant crops. The dots in (a–d) represent the combination of costs *ϕ*_*T*_ and *ϕ*_*R*_ that lie along the Pareto front in (e), and all were parameter combinations that disincentivize the use of the tolerant crop. (e) Pareto front when both the tolerant and the resistant crop were only moderately effective. The individual grey dots correspond to all pairs of values of (*ϕ*_*T*_, *ϕ*_*R*_) considered in our two‐way scan; the coloured dots are those that lay on the Pareto front. The darker the dots, the more the low costs to the planner have been prioritized. The Gini coefficients are shown for the fairest scenario (*G* = 0.009, when *τ* = 0.13 and P = 0.93; ‘X’ on the graphs) and the least fair scenarios (*G* = 0.5; ‘O’ when the profit is prioritized and ‘*’ when the costs to the planner are prioritized). (f) Pareto dominance between cost to the planner *τ* = 0.06 and 0.0, which corresponds to the ‘break’ in the Pareto front in (e). Other than the parameters being scanned over, and *δ*_*ιT*_ = 0.5, parameters and initial conditions are as in Tables 1 and 3. [Colour figure can be viewed at wileyonlinelibrary.com]

Here, as an example, we calculate the Pareto front when neither improved crop type is very effective (the relative susceptibility of the resistant crop is *δ*_*βR*_ = 0.5 and the relative loss due to disease in the tolerant crop is *δ*_*ιT*_ = 0.5). Figure 6 shows the Pareto front when the planner is trying to minimize the cost of the subsidy scheme while also maximizing the average profit of growers, with both quantities considered at the eventual equilibrium of the model.

We plotted the Pareto front onto a two‐way scan of the cost of the resistant and the tolerant crop (*ϕ*_*R*_ and *ϕ*_*T*_, respectively) to investigate the subsidy regime that produces optimal outcomes (Figure 6a–d). The strategies that guarantee the best outcome for both the growers and the planner were to use any combination of *ϕ*_*R*_ and *ϕ*_*T*_ that discourages widespread use of the tolerant crop (Figure 6d, where the Pareto front lies along the edge of the ‘U + T’ and the ‘U + R’ equilibrium). Below the value of *ϕ*_*R*_ = 0.365, the optimal subsidy schemes ensure a mixed equilibrium of unimproved and resistant crops (Figure 6d). Above this value (for which the only optimal strategies were *ϕ*_*T*_ = 0.4, *ϕ*_*R*_ = 0.365 or *ϕ*_*T*_ = 0.4, *ϕ*_*R*_ = 0.367), the resistant crop is too expensive and growers use the tolerant crop instead, though it is always at relatively low levels (around 10% of fields were planted with the tolerant crop, Appendix S3, Figure 4).

Though the use of a tolerant crop earns the growers high profits, the resulting increase in infection pressure induces a positive feedback loop that incentivizes other growers to also use the tolerant crop. Therefore, the planner had to subsidize more growers, meaning it is ultimately not economical for the planner, and no Pareto‐optimal strategy lies in the region where there is high use of the tolerant crop. Conversely, as the resistant crop provides benefit to growers who do not use it (i.e., free ride off the efforts of others), by subsidizing a minority of growers to use a resistant crop, the planner can achieve good profit outcomes averaged over the population as a whole for lower costs.

Though the Pareto front lies along the diagonal, any points in a given row to the right of the diagonal have the same value. This is because there is no tolerant crop at equilibrium, so changing the cost of the tolerant crop has no effect on the equilibrium achieved. Similarly, all points in a given column above the diagonal have the same value as there is no resistant crop at equilibrium. Therefore, these points are all considered to be Pareto optimal.

We then plotted the Pareto front for each combination of costs and profits from Figure 6a,c,e. The grey dots are subsidy strategies that were dominated by other strategies that lie on the Pareto front. The Pareto front is broken between *τ* = 0.058 and *τ* = 0.015 (indicated by green circles in Figure 6f). These combinations of costs and profits were dominated by *τ* = 0.015 and *P* = 0.88, where the profit is at a local maximum. The profits earned by growers when 0.058 > *τ* > 0.016 are all *P* < 0.88, so both growers and planners can achieve better outcomes at *τ* = 0.015. Between 0.058 > *τ* > 0.016, the subsidization scheme reduces use of the resistant crop, decreasing profits for the growers as more fields become infected.

The degree of subsidization (and associated outcome) depends on the planner's weighting of the relative importance of the cost to the planner and the profit of growers. This can be measured using Gini coefficients. The Gini coefficients are shown for the extremes of equality: the least‐fair scenarios, with a Gini coefficient of 0.5, lie at either end of the Pareto front. Time courses for these different scenarios are shown in Figure 3 in Appendix S5.

When the profit to growers is 1.00 and the cost to the planner is 0.25, corresponding to *ϕ*_*R*_ = 0.0 and *ϕ*_*T*_ = 0.05 (marked ‘O’ in Figure 6), a relatively high proportion of growers use the resistant crop (≈49.8%, Figure 4b in Appendix S5). At the other end of the front, the profit to the growers is 0.85 and *τ* = 0. As *ϕ*_*R*_ = 0.4 and *ϕ*_*T*_ = 0.375 (marked ‘*’ in Figure 6), so the improved crop is effectively not subsidized. This results in no users of the resistant crop and only around 10% of growers using the tolerant crop (Figure 3c in Appendix S5), reducing the costs to the planner (this is also the only point on the Pareto front where any growers use the tolerant crop; Figure 4a in Appendix S5).

The fairest scenario (G = 0.009, ‘X’) occurs when *ϕ*_*T*_ = 0.12 and *ϕ*_*R*_ = 0.105 (*τ* = 0.13 and *P* = 0.93). At this point, ≈40.3% of growers use the resistant crop, none use the tolerant crop (Figure 4 in Appendix S5), and only 6% of fields are infected (Figure 6b). In this ‘U + R’ equilibrium, the majority of growers can ‘free ride’ off the efforts of those using the resistant crop.

### 3.4 Effect of time on the Pareto front

Both the costs to the planner and the profits of growers depend on the proportion of fields of each type in the system. This has a strong temporal component; consequently, the Pareto front may change depending on the time horizon examined. We investigated this temporal effect after 1, 2, 3, 4 and 5 seasons, fixing the cost of the resistant crop (*ϕ*_*R*_) to 0.1 for ease of analysis (i.e., in this section, we only consider changes to the cost of the tolerant crop, *ϕ*_*T*_).

**FIGURE 7:**
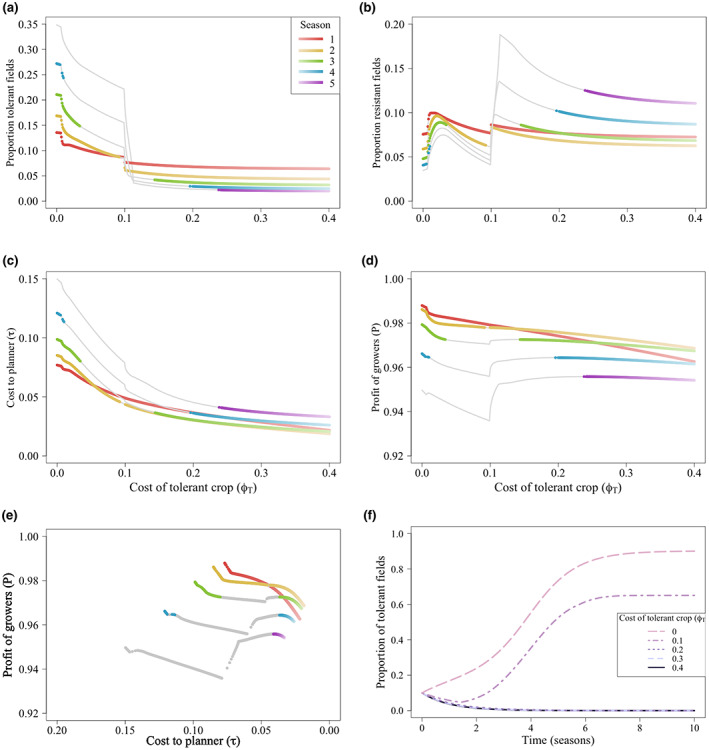
Sets of solutions with equally good outcomes (Pareto fronts) for minimizing the cost of a subsidy scheme while maximizing the average profit of growers for different growing seasons when both the tolerant and the resistant crops were only moderately effective. In different seasons, (a) the proportion of tolerant fields, (b) proportion of resistant fields, (c) cost of the subsidization scheme to the planner and (d) profit to the growers with increasing cost of the tolerant crop. The darker the colour, the more the profit of growers is prioritized over the cost to planners. Generally, the Pareto front lies along values of costs of the tolerant crop, *ϕ*_*T*_, that reduce the proportion of growers using the tolerant crop and increase those using the resistant crop. (e) At low time points, all subsidization schemes are Pareto optimal, although as seasons progress, there is a narrower range of optimal solutions. (f) Once *ϕ*_*T*_ > 0.2, no growers use the tolerant crop after five seasons. Other than *δ*_*ιT*_ = 0.5 and *δ*_*βR*_ = 0.5, parameters are as in Table 2. Note, in each of these graphs, the cost of the resistant crop (*ϕ*_*R*_) is 0.1. [Colour figure can be viewed at wileyonlinelibrary.com]

At short time points (one or two seasons), nearly every subsidization scheme lies on the Pareto front (Figure 7a–d). Each of these values of *ϕ*_*T*_ was considered equally efficient, and produces a Pareto‐optimal combination of outcomes for both the growers and the planners.

As the epidemic progresses (seasons 3, 4 and 5), only higher values of *ϕ*_*T*_ give Pareto‐optimal solutions (Figure 7a–d). The higher cost of the tolerant crop disincentivizes its use, so fewer growers use it (Figure 7a). Although there is an increase in the proportion using the resistant crop, its uptake is still relatively low (Figure 7b) due to the ability of non‐controllers to free ride. Overall, the higher values of *ϕ*_*T*_ lower the costs to the planner while maintaining high profits for the growers (Figure 7c–e).

However, between seasons 3 and 4 of the epidemic, low values of *ϕ*_*T*_ also permit Pareto‐optimal solutions. At these values, relatively few of the growers are using the tolerant crop (Figure 7f) and the costs to the planner are relatively low, while the profits to the grower are high (Figure 7c,d).

The Pareto fronts show that, over these shorter time horizons, there is often a small difference in the profits and costs for the optimal cases (Figure 7e). We again find that the costs of an improved crop that discourage the use of a tolerant crop were optimal. For each time horizon, the Pareto optimum is achieved at higher values of the cost of a tolerant crop to growers (*ϕ*_*T*_), which discourages its use. This implies that in the early stage of an epidemic, there is not a severe trade‐off between the optimal outcomes for the planner and the grower.

Figure 7f shows a time course of the proportion of growers using the tolerant crop. At low costs of the tolerant crop (*ϕ*_*T*_), which were only part of the Pareto‐optimal sets for short time horizons (between one and four seasons), use of this tolerant crop does not die out. At higher values, use dies out within five seasons. Therefore, while lower values of *ϕ*_*T*_ can sometimes provide Pareto‐optimal solutions, higher values give optimal outcomes irrespective of the time horizon.

The same results hold for other parameterizations of tolerance and resistance—irrespective of how good or bad the improved crop varieties are, the Pareto‐optimal strategies were those in which the use of the tolerant crop is discouraged.

## 4 DISCUSSION

Human behaviour has increasingly been investigated in the context of plant disease epidemiology (Bate et al., 2021; McQuaid et al., 2017; Milne et al., 2016, 2020; Murray‐Watson et al., 2022; Murray‐Watson & Cunniffe, 2022; Saikai et al., 2021), where models allow growers to choose between engaging in disease control or taking no action. The proportion of growers using control depends on the control strategy itself, with previous studies investigating the uptake of clean seed systems among cassava growers (McQuaid et al., 2017; Murray‐Watson et al., 2022), transgenic herbicide‐resistant maize (Milne et al., 2016; Saikai et al., 2021) and disease‐tolerant or ‐resistant crops (Murray‐Watson & Cunniffe, 2022). Uptake will also depend on the relative costs of control and losses due to disease, among other factors not investigated here (such as the social connections between growers). However, in each of these examples, growers could only choose whether to use control or not, not between different types of control.

When investigating the effect of having crop that was either tolerant or resistant to TYLCV available to growers (alongside an unimproved crop), Murray‐Watson and Cunniffe (2022) found that widespread use of a tolerant crop was often achieved. A tolerant crop only benefited those growers using it, and due to the lower rate of removal via roguing, they increased the infection pressure on other growers (generating negative externalities). However, when a resistant crop was available, such high levels of adoption were not achieved. A resistant crop protected other unimproved fields (generating positive externalities), who can free ride off the efforts of those using the resistant crop.

These results were corroborated in the work presented here. Even when growers have a choice between three crop types (unimproved, disease‐tolerant or disease‐resistant), the positive feedback loop induced by the use of a tolerant crop is sufficient to ensure that growers will overwhelmingly use the tolerant crop, even when it is ineffective (Figure 4a). This positive feedback loop means that a tolerant crop is a ‘strategic complement’ (a strategy that incentivizes others to adopt the strategy as more individuals use that strategy; Hennessy, 2008). A resistant crop, by contrast, is a ‘strategic substitute’: its use discourages others from also using a resistant crop, as they can benefit from the efforts of others.

With the default costs of each crop type (where the grower paid *ϕ*_*T*_ = *ϕ*_*R*_ = 0.1), only when the tolerant crop is very ineffective (sustaining a high relative loss of yield) do some growers consider the resistant crop (Figure 4a). Similarly, if the resistant crop is very effective (with a low relative susceptibility), there will be a mixed ‘resistant and unimproved’ crop equilibrium (Figure 5). However, due to the free riding by other growers, universal adoption of the resistant crop is not achieved, echoing an analogous result in the simpler, two‐strategy model (Murray‐Watson & Cunniffe, 2022). Therefore, a mixed equilibrium persists (such as the ‘unimproved and resistant’ equilibrium in Figure 5a).

We used the concept of Pareto efficient strategies (Luc, 2008) to optimize the dual objectives of ensuring high profits for growers while also minimizing the cost to social planners. The positive feedback induced by the use of a tolerant crop means that it will have widespread adoption by growers, so those providing subsidies would have to do so for nearly all growers. This increases the cost to the planner (*τ*), though it does increase the profits to the growers (*P*) (Figure 6e). However, relatively high profits can also be obtained when a resistant crop is subsidized (at least 85% of the maximum theoretical profit, Figure 6e). Furthermore, as the adoption of the resistant crop is not as widespread as that of the tolerant crop, most of the profits can be achieved with less investment from the planner. Therefore, the control strategies on the Pareto front require the planner to provide sufficient subsidies to the resistant crop to ensure that it is used, while never incentivizing the tolerant crop (Figure 6a–d). Most optimal subsidization schemes result in an ‘unimproved and resistant’ crop equilibrium (Figure 6d).

By targeting subsidies at the resistant crop, the social planner can exploit the free riding behaviour of growers using the unimproved crop and only subsidize a subset of growers. Targeting subsidies to the resistant crop can be seen as a way of internalizing some of the positive externalities produced by these growers; conversely, not subsidizing the tolerant crop internalizes their negative externalities (Gersovitz, 2014). This strategy was optimal, irrespective of the time course over which profits and costs were calculated (Figure 7f). However, earlier in the epidemic (between seasons 1 and 4), subsidization schemes that resulted in some fields planted with the tolerant crop were possible, but only when the costs to the planner were sufficiently low (Figure 7).

These models ignore the possibility of virus evolution, which is a major threat to the durability of genetic disease control mechanisms (Gallois et al., 2018; Sett et al., 2022). Tolerant crops, by allowing pathogen replication, do not exert the same selection pressure (Råberg et al., 2009) and, therefore, may provide a more durable protection against yield loss. Indeed, in van den Bosch et al. (2006), the authors defined four types of resistance, one of which—‘symptom‐reducing resistance’—is similar in mechanism to what we define as tolerant in this article. The authors note that this form of resistance does not put pressure on the pathogen to evolve higher mutation rates. Though we do not explore viral evolution in this article, it would be an interesting extension given its possible effects on the durability of the control schemes.

Resistant crops exert much stronger selection pressures on the pathogen, often leading to resistance breakdown where the crop's resistant traits become ineffective against infection (Parlevliet, 2002). Over the short term, it may be cheaper for the planner to subsidize the use of resistant crops, but when considering the potential evolution of a resistance‐breaking pathogen and the associated costs of developing and disseminating new crop types, a tolerant crop may be more favourable.

Other simplifying assumptions were made for this study. We did not include spatial or stochastic effects (Fabre et al., 2021), both of which can influence disease progression and growers' decision‐making. We also did not differentiate between the nature or quantity of information available to each grower, both of which will depend on the grower's social and professional network (Milne et al., 2016; Sherman & Gent, 2014), or allow for differing perceptions of risk (our values of responsiveness, *η*_*b*_, *b* ∈ {*U*, *T*, *R*} were the same across all growers irrespective of control strategy). A grower's risk attitude will be influenced by factors such as their previous experience with disease outbreaks or received knowledge from other growers (Sherman & Gent, 2014) and will impact their control decisions and consequently disease progression (Murray‐Watson et al., 2022). Growers, then, are ultimately ‘reflexive producers’ (Kaup, 2008), and their control decisions must balance each of these information sources with external factors such as market pressures.

The decision to model just three alternatives of the same crop was simplification. In reality, if there was a very high disease prevalence, or all crop varieties proved unprofitable, it is possible that a grower would consider an alternative crop entirely. In addition, we have simplified the range of control options available to growers. We have focused on two improved crop varieties, but in reality, the growers would have access to a broader range of varieties with different traits and are likely to employ other mechanisms as part of an IPM scheme. This may include silver mulch, insecticides or protective nets (Riley & Srinivasan, 2019). Growers of resistant crops, who risk higher yield losses, may also be more likely to engage in such IPM practices.

Our model demonstrates how decision models of grower behaviour can be combined with other economic concepts, such as Pareto fronts, to find socially optimal solutions across conflicting objectives. We found that what was optimal for the growers (using a tolerant crop) led to worse outcomes for the planner, and the social optimum occurred when a subset of the growers used a resistant crop. Though we examine these trade‐offs for a crop that is either tolerant or resistant to TYLCV, the model form is sufficiently flexible to allow for other pathosystems or control mechanisms.

### OPEN RESEARCH BADGES

This article has earned an Open Data badge for making publicly available the digitally‐shareable data necessary to reproduce the reported results. The data is available at https://github.com/RachelMurray‐Watson/Expanding‐growers‐choice‐of‐disease‐management‐options‐can‐promote‐suboptimal‐social‐outcomes.git.

## Supporting information


Appendix S1.

Appendix S2.

Appendix S3.

Appendix S4.

Appendix S5.

## Data Availability

Base code is available at: https://github.com/RachelMurray‐Watson/Expanding‐growers‐choice‐of‐disease‐management‐options‐can‐promote‐suboptimal‐social‐outcomes.git (Murray‐Watson, 2022).
