# Supplementary material for: Expanding growers' choice of plant disease management options can promote suboptimal social outcomes
Source: Plant Pathol. 2023 Feb 6;72(5):933–50. doi: 10.1111/ppa.13705 (PMC10952642; doi:10.1111/ppa.13705)
Supplement: Supplementary file 1 — Appendix S1. [file PPA-72-933-s001.pdf]

7 Appendix 1: Calculating the expected profit

To calculate the expected profit, we must first calculate the profits associated with each possible outcome a grower could achieve at the time of harvest (or when a field is rogued). These will depend on the control strategy used by the grower (whether they planted tolerant, resistant, or unimproved crop at the beginning of the season), the infectious status of their field and whether an infectious field was rogued.

The profits for each field type are given as follows:

$$
\begin{aligned}
P_{SU} &= \text{Profit for non-controller with a susceptible field,} \\
&= Y, && (42) \\
P_{EU} &= \text{Profit for non-controller with latently-infected field,} \\
&= Y, && (43) \\
P_{I_H U} &= \text{Profit for non-controller with an infected field that was not rogued,} \\
&= \psi - L, && (44) \\
P_{I_R U} &= \text{Profit for non-controller with an infected field that was rogued,} \\
&= \psi - \phi_Q L, && (45) \\
P_{ST} &= \text{Profit for controller using tolerant crop with a susceptible field,} \\
&= \delta_{\psi_T}\psi - \phi_T, && (46) \\
P_{ET} &= \text{Profit for controller using tolerant crop with latently-infected field,} \\
&= \delta_{\psi_T}\psi - \phi_T, && (47) \\
P_{I_H T} &= \text{Profit for controller using tolerant crop with an infected field that was not rogued,} \\
&\quad = \delta_{\psi_T}\psi - \phi_T - \iota_T, && (48) \\
P_{I_R T} &= \text{Profit for controller using tolerant crop with an infected field and that was rogued,} \\
&\quad = \delta_{\psi_T}\psi - \phi_T - \phi_Q \iota_T. && (49) \\
P_{SR} &= \text{Profit for controller using resistant crop with a susceptible field,} \\
&= \delta_{\psi_R}\psi - \phi_R, && (50) \\
P_{ER} &= \text{Profit for controller using resistant crop with latently-infected field,} \\
&= \delta_{\psi_R}\psi - \phi_R, && (51) \\
P_{I_H R} &= \text{Profit for controller using resistant crop with an infected field that was not rogued,} \\
&\quad = \delta_{\psi_R}\psi - \phi_R - \iota_R, && (52) \\
P_{I_R R} &= \text{Profit for controller using resistant crop with an infected field and that was rogued,} \\
&\quad = \delta_{\psi_R}\psi - \phi_R - \phi_Q \iota_R. && (53)
\end{aligned}
$$

The expected profits for each strategy depend on the probabilities of a grower receiving each of these payoffs.

For each crop variety, the probability of infection (q_j) is given as:

$$
\begin{aligned}
q_j = & \text{ Instantaneous probability of horizontal infection,} \\
= & \frac{\text{Instantaneous infection rate}}{\text{Instantaneous infection rate + Harvesting rate}},
\end{aligned}
$$

so

$$
q_U = \frac{\beta(I_U + \delta_{\sigma_T} I_T + \delta_{\sigma_R} I_R)}{\beta(I_U + I_T + \sigma I_U) + \gamma}, \tag{54}
$$

$$
q_T = \frac{\delta_{\beta_T}\beta(I_U + \delta_{\sigma_T} I_T + \delta_{\sigma_R} I_R)}{\delta_{\delta_{\beta_T}}\beta(I_U + I_T + \sigma I_U) + \gamma}, \tag{55}
$$

$$
q_R = \frac{\delta_{\delta_{\beta_R}}\beta(I_U + \delta_{\sigma_T} I_T + \delta_{\sigma_R} I_R)}{\delta_{\delta_{\beta_R}}\beta(I_U + I_T + \sigma I_U) + \gamma}. \tag{56}
$$

The expected profit, however, will also be dependent on whether a grower's field is harvested whilst latently infected (q_{Eb}) or proceeds to become fully infectious (q_{Ib}). The probability of being harvested whilst latently infected is given as:

$$
q_{EU} = \frac{\gamma}{\epsilon + \gamma}, \tag{57}
$$

$$
q_{ET} = \frac{\gamma}{\delta_{\epsilon_T}\epsilon + \gamma}, \tag{58}
$$

$$
q_{ER} = \frac{\gamma}{\delta_{\epsilon_R}\epsilon + \gamma}, \tag{59}
$$

and the probability of becoming fully infectious before harvest is:

$$q_{IU} = \frac{\epsilon}{\epsilon + \gamma}, \tag{60}$$

$$q_{IT} = \frac{\delta_{\epsilon_T}\epsilon}{\delta_{\epsilon_T}\epsilon + \gamma}, \tag{61}$$

$$q_{IR} = \frac{\delta_{\epsilon_R}\epsilon}{\delta_{\epsilon_R}\epsilon + \gamma}. \tag{62}$$

Fully infectious crops can either be harvested or removed via roguing. The probability that harvesting occurs before roguing ($q_{I_H b}$) is given by:

$$q_{I_H b} = q_{Ib}\left(\frac{\gamma}{\gamma + \mu_b}\right), \tag{63}$$

and that it is rogued before harvesting ($q_{I_R b}$) is:

$$q_{I_R b} = q_{Ib}\left(\frac{\mu_b}{\gamma + \mu_b}\right), \tag{64}$$

We can then use these probabilities to calculate the expected profit of each strat-

egy:

$$
\begin{aligned}
P_U &= \text{Grower's estimate of the expected profit next season if control is not adopted,} \\
&= (1-q_U)P_{SU} + q_{EU}P_{EU} + q_{I_HU}P_{I_HU} + q_{I_RU}P_{I_RU}, \qquad (65) \\
P_T &= \text{Grower's estimate of the expected profit next season if tolerant crop is used,} \\
&= (1-q_T)P_{ST} + q_{ET}P_{ET} + q_{I_HT}P_{I_HT} + q_{I_RT}P_{I_RT}, \qquad (66) \\
P_R &= \text{Grower's estimate of the expected profit next season if resistant crop is used,} \\
&= (1-q_R)P_{SR} + q_{ER}P_{ER} + q_{I_HR}P_{I_HR} + q_{I_RR}P_{I_RR}. \qquad (67)
\end{aligned}
$$

We can simplify these expressions to:

$$
\begin{aligned}
P_U &= \psi - q_U \frac{\epsilon}{\epsilon+\gamma}\iota\left(\frac{\gamma}{\gamma+\mu_U} + \frac{\mu_U}{\mu_U+\gamma}\phi_Q\right), \qquad (68) \\
P_T &= \delta_{\psi_T}\psi - \phi_T - q_T \frac{\delta_{\epsilon_T}\epsilon}{\delta_{\epsilon_T}\epsilon+\gamma}\delta_{\iota_T}L\left(\frac{\gamma}{\gamma+\mu_T} + \frac{\mu_T}{\mu_T+\gamma}\phi_Q\right), \qquad (69) \\
P_R &= \delta_{\psi_R}\psi - \phi_R - q_R \frac{\delta_{\epsilon_R}\epsilon}{\delta_{\epsilon_R}\epsilon+\gamma}\delta_{\iota_R}\iota\left(\frac{\gamma}{\gamma+\mu_R} + \frac{\mu_R}{\mu_R+\gamma}\phi_Q\right). \qquad (70)
\end{aligned}
$$
