# Supplementary material for: Expanding growers' choice of plant disease management options can promote suboptimal social outcomes
Source: Plant Pathol. 2023 Feb 6;72(5):933–50. doi: 10.1111/ppa.13705 (PMC10952642; doi:10.1111/ppa.13705)
Supplement: Supplementary file 2 — Appendix S2. [file PPA-72-933-s005.pdf]

8 Appendix 2: Details of switching terms

The switching terms take the general form: a grower with outcome P_{ab}, is switching into strategy c or d, $a \in \{S, E, I_H, I_R\}$ and $b, c, d \in \{U, T, R\}, b \neq c \neq d$. If the profits of both c and d are exactly equal, the grower will switch into whichever strategy has the higher proportion of current users. If both the expected profits and the proportion of growers using each strategy are the same, then half of the growers considering changing strategy will compare with each alternative expected profit.

The full details of all the switching terms are given below. If $P_T < P_R$ or $P_T = P_R$ and $R > T$ (i.e. there are more using resistant than tolerant crop), then

$$z_{SUR} = \max(0, 1 - \exp(-\eta(P_R - P_{SU})), \tag{71}$$

$$z_{EUR} = \max(0, 1 - \exp(-\eta(P_R - P_{EU})), \tag{72}$$

$$z_{I_HUR} = \max(0, 1 - \exp(-\eta(P_R - P_{I_HU})), \tag{73}$$

$$z_{I_RUR} = \max(0, 1 - \exp(-\eta(P_R - P_{I_RU})), \tag{74}$$

$$z_{SUT} = 0, \tag{75}$$

$$z_{EUT} = 0, \tag{76}$$

$$z_{I_HUT} = 0, \tag{77}$$

$$z_{I_RUT} = 0. \tag{78}$$

If $P_T > P_R$ or $P_T = P_R$ and $R < T$, then

$$z_{SUR} = 0, \tag{79}$$

$$z_{EUR} = 0, \tag{80}$$

$$z_{I_H UR} = 0, \tag{81}$$

$$z_{I_R UR} = 0, \tag{82}$$

$$z_{SUT} = \max(0, 1 - \exp(-\eta(P_T - P_{SU})) \tag{83}$$

$$z_{EUT} = \max(0, 1 - \exp(-\eta(P_T - P_{EU})) \tag{84}$$

$$z_{I_H UT} = \max(0, 1 - \exp(-\eta(P_T - P_{I_H U})) \tag{85}$$

$$z_{I_R UT} = \max(0, 1 - \exp(-\eta(P_T - P_{I_R U})). \tag{86}$$

If $P_T < P_U$ or $P_T = P_U$ and $U > T$, then

$$z_{SRU} = \max(0, 1 - \exp(-\eta(P_U - P_{SR})), \quad (87)$$

$$z_{ERU} = \max(0, 1 - \exp(-\eta(P_U - P_{ER})), \quad (88)$$

$$z_{I_H RU} = \max(0, 1 - \exp(-\eta(P_U - P_{I_H R})), \quad (89)$$

$$z_{I_R RU} = \max(0, 1 - \exp(-\eta(P_U - P_{I_R R})), \quad (90)$$

$$z_{SRT} = 0, \quad (91)$$

$$z_{ERT} = 0, \quad (92)$$

$$z_{I_H RT} = 0, \quad (93)$$

$$z_{I_R RT} = 0. \quad (94)$$

If $P_T > P_U$ or $P_T = P_U$ and $U < T$, then

$$z_{SRU} = 0, \tag{95}$$

$$z_{ERU} = 0, \tag{96}$$

$$z_{I_H RU} = 0, \tag{97}$$

$$z_{I_R RU} = 0, \tag{98}$$

$$z_{SRT} = \max(0, 1 - \exp(-\eta(P_T - P_{SR})), \tag{99}$$

$$z_{ERT} = \max(0, 1 - \exp(-\eta(P_T - P_{ER})), \tag{100}$$

$$z_{I_H RT} = \max(0, 1 - \exp(-\eta(P_T - P_{I_H R})), \tag{101}$$

$$z_{I_R RT} = \max(0, 1 - \exp(-\eta(P_T - P_{I_R R})). \tag{102}$$

If $P_U > P_R$ or $P_U = P_R$ and $R < U$, then

$$z_{STU} = \max(0, 1 - \exp(-\eta(P_U - P_{ST})), \tag{103}$$

$$z_{ETU} = \max(0, 1 - \exp(-\eta(P_U - P_{ET})), \tag{104}$$

$$z_{I_H TU} = \max(0, 1 - \exp(-\eta(P_U - P_{I_H T})), \tag{105}$$

$$z_{I_R TU} = \max(0, 1 - \exp(-\eta(P_U - P_{I_R T})), \tag{106}$$

$$z_{STR} = 0, \tag{107}$$

$$z_{ETR} = 0, \tag{108}$$

$$z_{I_H TR} = 0, \tag{109}$$

$$z_{I_R TR} = 0. \tag{110}$$

If $P_U < P_R$ or $P_U = P_R$ and $R > U$, then

$$z_{STU} = 0, \tag{111}$$

$$z_{ETU} = 0, \tag{112}$$

$$z_{I_HTU} = 0, \tag{113}$$

$$z_{I_RTU} = 0, \tag{114}$$

$$z_{STR} = \max(0, 1 - \exp(-\eta(P_R - P_{ST})), \tag{115}$$

$$z_{ETR} = \max(0, 1 - \exp(-\eta(P_R - P_{ET})), \tag{116}$$

$$z_{I_HTR} = \max(0, 1 - \exp(-\eta(P_R - P_{I_HT})), \tag{117}$$

$$z_{I_RTR} = \max(0, 1 - \exp(-\eta(P_R - P_{I_RT})). \tag{118}$$

In the case where both the profits and the proportion of growers using the alternative strategy are equal, growers changing strategy will be divided evenly between the two alternative strategies.

To simplify writing the model, we can then say:

$$z_{SU} = \max(z_{SUT}, z_{SUR}), \tag{119}$$

$$z_{EU} = \max(z_{EUT}, z_{EUR}), \tag{120}$$

$$z_{I_H U} = \max(z_{I_H UT}, z_{I_H UR}), \tag{121}$$

$$z_{I_R U} = \max(z_{I_R UT}, z_{I_R UR}), \tag{122}$$

$$z_{ST} = \max(z_{STU}, z_{STR}), \tag{123}$$

$$z_{ET} = \max(z_{ETU}, z_{ETR}), \tag{124}$$

$$z_{I_H T} = \max(z_{I_H TU}, z_{I_H TR}), \tag{125}$$

$$z_{I_R T} = \max(z_{I_R TU}, z_{I_R TR}), \tag{126}$$

$$z_{SR} = \max(z_{SRU}, z_{SRT}), \tag{127}$$

$$z_{ER} = \max(z_{ERU}, z_{ERT}), \tag{128}$$

$$z_{I_H R} = \max(z_{I_H RU}, z_{I_H RT}), \tag{129}$$

$$z_{I_R R} = \max(z_{I_R RU}, z_{I_R RT}). \tag{130}$$
