# Supplementary material for: Expanding growers' choice of plant disease management options can promote suboptimal social outcomes
Source: Plant Pathol. 2023 Feb 6;72(5):933–50. doi: 10.1111/ppa.13705 (PMC10952642; doi:10.1111/ppa.13705)
Supplement: Supplementary file 3 — Appendix S3. [file PPA-72-933-s003.pdf]

9 Appendix 3: Calculating the basic reproduction number for the model

At the disease-free equilibrium, if growers have a choice between strategies they would never chose to pay for control. Thus, in the behavioural model, the disease-free equilibrium is given as: $(S_U, E_U, I_U, S_T, E_T, I_T, S_R, E_R, I_R) = (N, 0, 0, 0, 0, 0, 0, 0, 0)$.

For this model, the transmission matrix F is given by: $F =$

$$F = \begin{bmatrix} 0 & \beta_U N & 0 & \beta_U N & 0 & \sigma\beta_U N \\ 0 & 0 & 0 & 0 & 0 & 0 \\ 0 & 0 & 0 & 0 & 0 & 0 \\ 0 & 0 & 0 & 0 & 0 & 0 \\ 0 & 0 & 0 & 0 & 0 & 0 \\ 0 & 0 & 0 & 0 & 0 & 0 \end{bmatrix}, \tag{131}$$

and the transition matrix, V, is given by:

$$V = \begin{bmatrix} \epsilon+\gamma & 0 & 0 & 0 & 0 & 0 \\ -\epsilon & \mu_U+\gamma & 0 & 0 & 0 & 0 \\ 0 & 0 & \delta_{\epsilon_T}\epsilon+\gamma & 0 & 0 & 0 \\ 0 & 0 & -\delta_{\epsilon_T}\epsilon & \mu_T+\gamma & 0 & 0 \\ 0 & 0 & 0 & 0 & \delta_{\epsilon_R}\epsilon+\gamma & 0 \\ 0 & 0 & 0 & 0 & -\delta_{\epsilon_R}\epsilon & \mu_R+\gamma \end{bmatrix}. \tag{132}$$

The inverse of V is given by:

$$V^{-1} = \begin{bmatrix} \frac{1}{\epsilon+\gamma} & 0 & 0 & 0 & 0 & 0 \\ \frac{\epsilon}{(\epsilon+\gamma)(\mu_U+\gamma)} & \frac{1}{\mu_U+\gamma} & 0 & 0 & 0 & 0 \\ 0 & 0 & \frac{1}{\delta_{\epsilon_T}\epsilon+\gamma} & 0 & 0 & 0 \\ 0 & 0 & \frac{\delta_{\epsilon_T}\epsilon}{(\delta_{\epsilon_T}\epsilon+\gamma)(\mu_T+\gamma)} & \frac{1}{\gamma+\mu_T} & 0 & 0 \\ 0 & 0 & 0 & 0 & \frac{1}{\delta_{\epsilon_R}\epsilon+\gamma} & 0 \\ 0 & 0 & 0 & 0 & \frac{\delta_{\epsilon_R}\epsilon}{(\delta_{\epsilon_R}\epsilon+\gamma)(\gamma+\mu_R)} & \frac{1}{\mu_R+\gamma} \end{bmatrix}. \tag{133}$$

The NGM, $K = FV^{-1}$, is then given by:

$$K = \begin{bmatrix} \frac{\beta_U \epsilon N}{(\epsilon+\gamma)(\mu_U+\gamma)} & \frac{\beta_U N}{\mu_U+\gamma} & \frac{\beta_U \delta_{\epsilon_T} \epsilon N}{(\delta_{\epsilon_T}\epsilon+\gamma)(\mu_T+\gamma)} & \frac{\beta_U N}{\mu_T+\gamma} & \frac{\beta_U \delta_{\epsilon_R} \epsilon \sigma N}{(\delta_{\epsilon_R}\epsilon+\gamma)(\mu_R+\gamma)} & \frac{\beta_U \sigma N}{\mu_R+\gamma} \\ 0 & 0 & 0 & 0 & 0 & 0 \\ 0 & 0 & 0 & 0 & 0 & 0 \\ 0 & 0 & 0 & 0 & 0 & 0 \\ 0 & 0 & 0 & 0 & 0 & 0 \\ 0 & 0 & 0 & 0 & 0 & 0 \end{bmatrix}. \quad (134)$$

The leading eigenvalue for this matrix is

$$R_0 = \frac{\beta_U \epsilon N}{(\epsilon + \gamma)(\mu_U + \gamma)}. \quad (135)$$
