# Supplementary material for: Expanding growers' choice of plant disease management options can promote suboptimal social outcomes
Source: Plant Pathol. 2023 Feb 6;72(5):933–50. doi: 10.1111/ppa.13705 (PMC10952642; doi:10.1111/ppa.13705)
Supplement: Supplementary file 4 — Appendix S4. [file PPA-72-933-s004.pdf]

10 Appendix 4: Sensitivity Scan

To investigate the effect of our choice of default parameters on the final equilibrium attained, we conducted additional parameter scans over parameters related to tolerance and resistance (δ_{ι_T} and δ_{ν_T} for tolerance, and δ_{ϵ_R}, δ_{β_R}, and δ_{σ_R} for resistance) (Figure 1). We also investigated the effect of changing the reduction in the loss due to disease when a field is rogued (ϕ_Q).

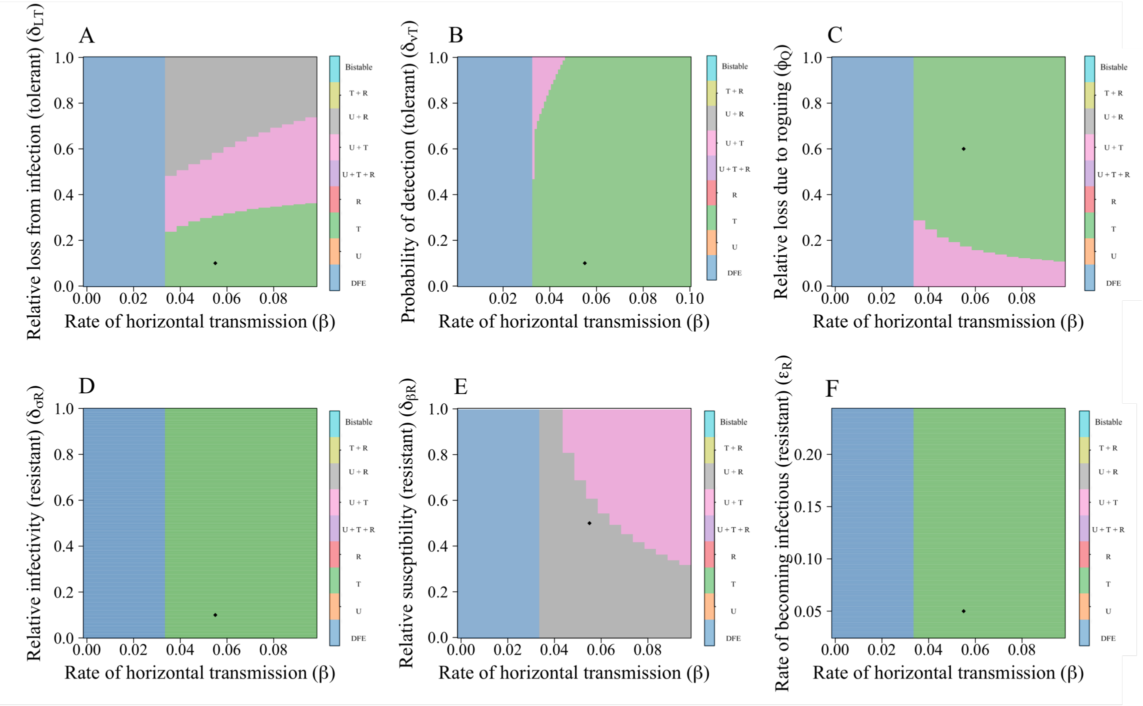

Figure 1: **Sensitivity scan of parameters relating to tolerance and resistance.** The possible equilibria attained when a two-way parameter scan is conducted between the rate of horizontal transmission and (A) the relative loss due to infection in tolerant crop (δ_{ι_T}), (B) the probability of detection in tolerant crop (δ_{ν_T}), (C) the reduction in the loss due to disease when a field is rogued (ϕ_Q), (D) the relative infectivity of resistant crop (δ_{σ_R}), (E) the relative susceptibility of resistant crop (δ_{β_R}) and (F) the rate of the development of infectivity and symptoms in resistant crop (ϵ_R). Black dots show the default parameterisation.

We can see that the parameter values do have an impact on the possible equilibria that are attained. Generally, the better the tolerant crop (in terms of its loss due to disease, δ_{ι_T}, and its detectability, δ_{ν_T}), the broader the range of equilibrium values where the tolerant crop was present at equilibrium. The same is true for the respective parameters for the resistant crop.

Figures 1 (D) and (F) are identical as, because of the background parameterisation, the use of resistant crop is never favourable for these parameters. Even as resistance becomes more effective, growers should still use tolerant crop.
