# Supplementary material for: Expanding growers' choice of plant disease management options can promote suboptimal social outcomes
Source: Plant Pathol. 2023 Feb 6;72(5):933–50. doi: 10.1111/ppa.13705 (PMC10952642; doi:10.1111/ppa.13705)
Supplement: Supplementary file 5 — Appendix S5. [file PPA-72-933-s002.pdf]

11 Appendix 5: Supplementary results

11.1 Change in switching points for parameter scans

The underlying bistability of the model means that, for a given set of parameters, different equilibria can be attained depending on the initial conditions. This manifests in our parameter scans over the relative loss due to disease in tolerant crop (δ_{ι_T}) and the relative susceptibility in resistant crop (δ_{β_R}) (Figures 4 and 5).

In these parameter scans, the initial conditions influence the parameter value at which the system switches from a mixed "tolerant and unimproved crop" equilibrium to a "resistant and unimproved crop" equilibrium in the δ_{ι_T} parameter scan (and vice versa in the δ_{β_R} scan). When the switch occurs depends strongly on the initial proportion of tolerant crop (Figures 2 and 1, which show the "extreme" parameter values for which the switch can occur). Importantly, this does not change the parameter values where growers of infected tolerant crop should switch strategy, as this occurs within the range of δ_{ι_T} that is unaffected by bistability (Figure 1 (B) and (D)).

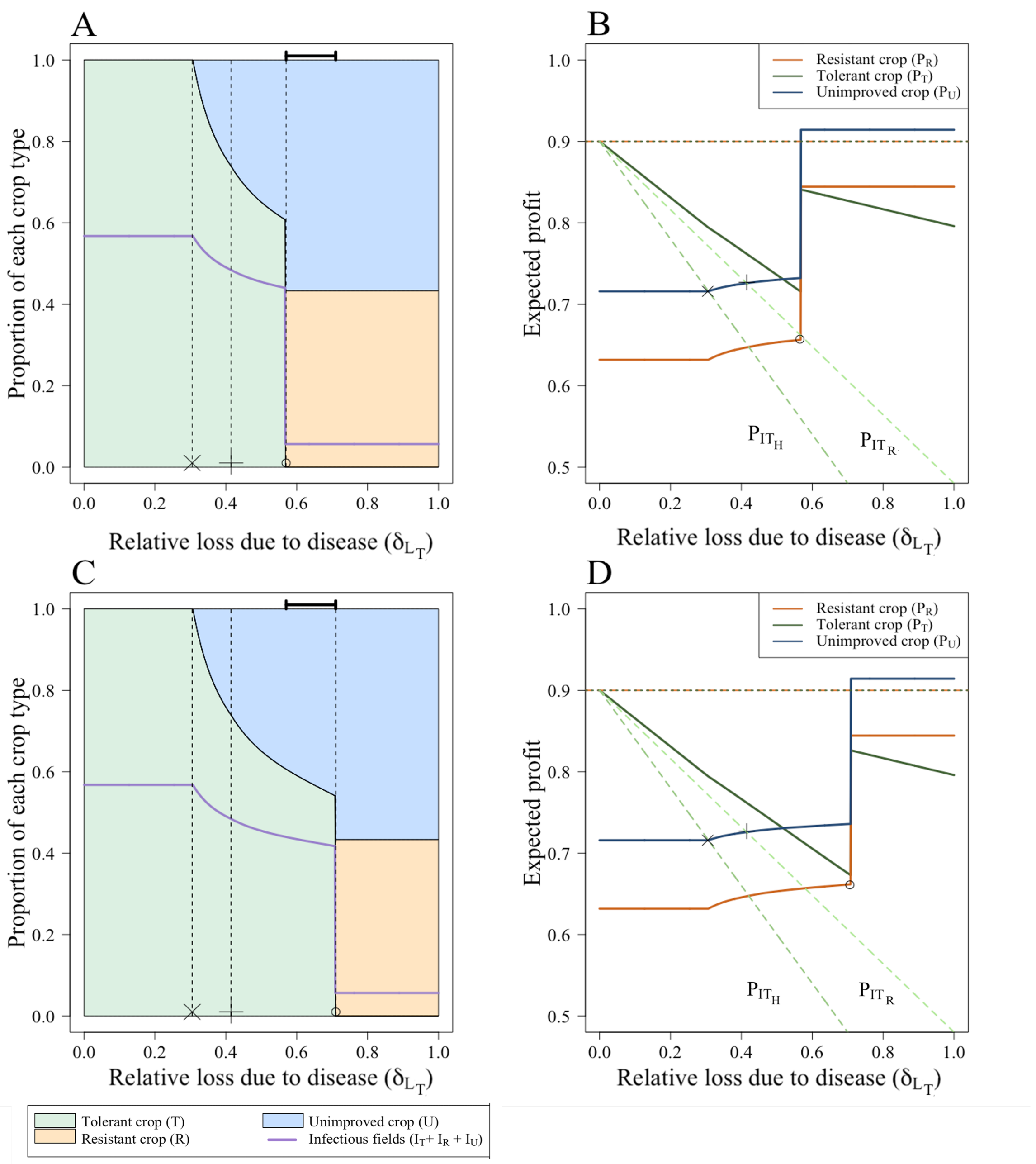

Figure 1: **Parameter scans showing range of δ_{ι_T} values during which switch in equilibria can occur.**(A) + (B) When none of the fields are initially planted with tolerant crop ($T_0 = 0$), the switch to the "resistant and unimproved" equilibrium occurs at $\delta_{\iota_T} = 0.51$ ("O"). (C) + (D) When all of the fields were initially planted with tolerant crop, the switch to the "resistant and unimproved" doesn't occur until $\delta_{\iota_T} = 0.51$ ("O").

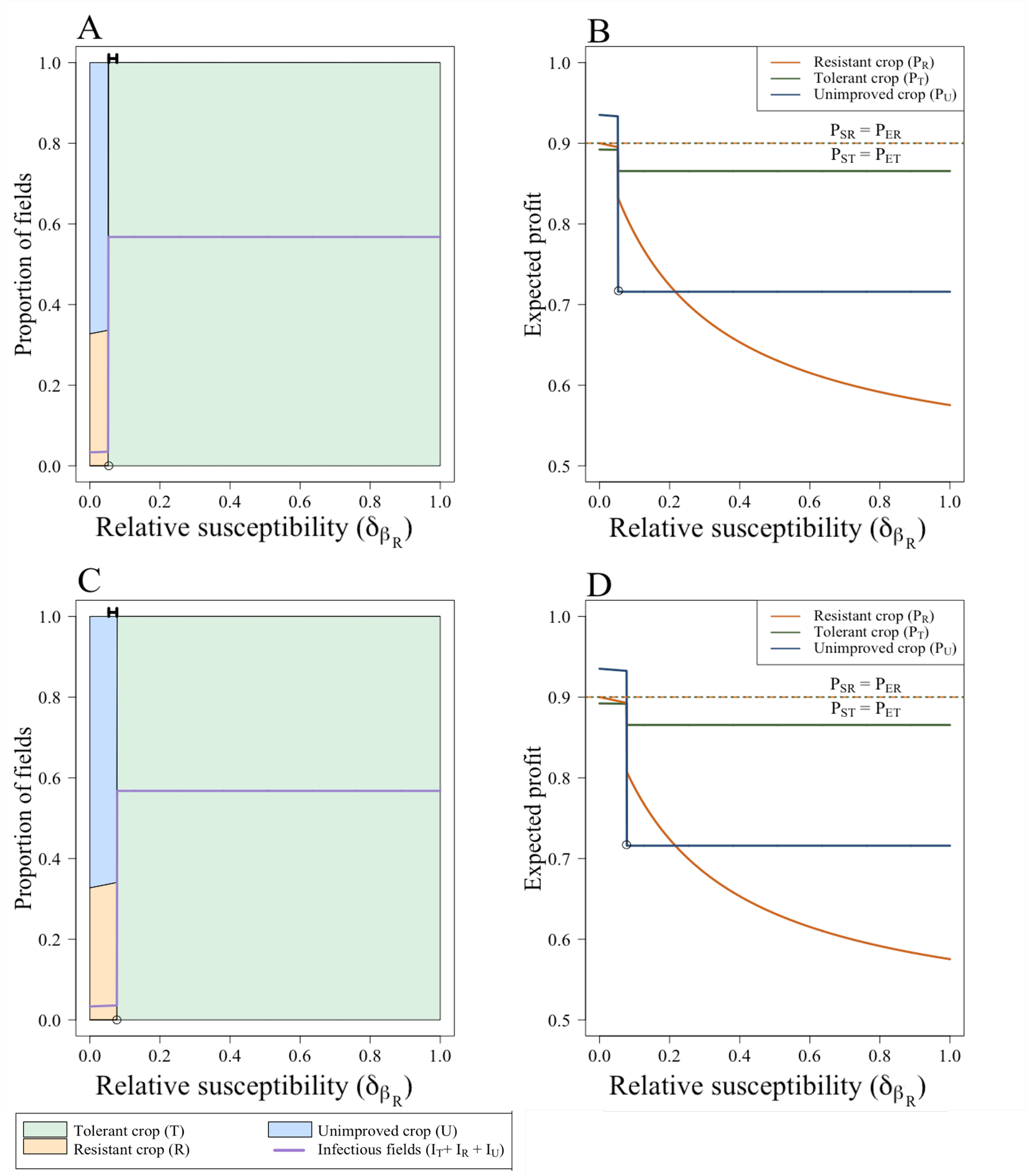

Figure 2: **Parameter scans showing range of δ_{β_T} values during which switch in equilibria can occur.** When none of the fields are initially planted with tolerant crop ($T_0 = 0$), the switch to the "resistant and unimproved crop" equilibrium occurs at a lower value of δ_{β_R}. When all of the fields are initially planted with tolerant crop ($T_0 = 0$; (A)), the switch to the "resistant and unimproved" equilibrium occurs at lower values of δ_{β_R} than if $T_0 = 1$; (B).

11.2 Time courses for fairest and least-fair subsidisation schemes

The Pareto front is bounded by two extremes: one where the profit of growers is maximised ($P = 1$), and the other when the cost to the planner is minimised ($\tau = 0$). Both of these scenarios have a Gini coefficient of 0.5. To prioritise the growers' profits, the cost of both resistant and tolerant crop must be low ($\phi_R = 0$ and $\phi_T = 0.05$). Conversely, to minimise the cost to the planner, both crops must be expensive for the growers ($\phi_R = 0.4$ and $\phi_T = 0.375$).

The fairest scenario ($G = 0.009$) occurs when $\tau = 0.13$ and $P = 0.93$. To achieve this, the cost of tolerant crop (ϕ_T) is 0.12, and the cost of resistant crop (ϕ_R) is 0.105.

Here, we show the dynamics of the model for each scenario. In both the "fairest" scenario and when the profits to the grower are preferred, the subsidisation scheme results in a high proportion using resistant crop and very low levels of disease (Figure 3(A)-(B)). When the costs to the planner are minimised, then no growers use resistant crop, and very few use tolerant crop (which is not subsidised, but still provides a reduced loss in yield if infected).

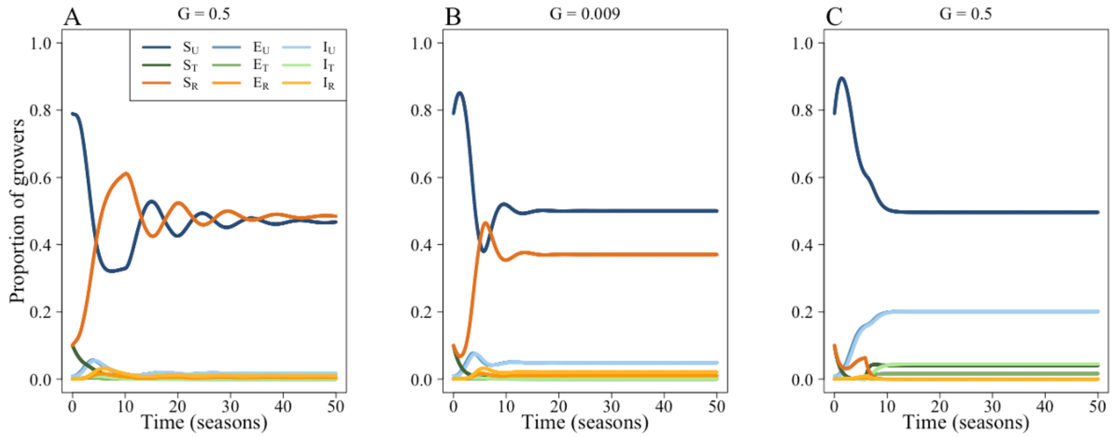

Figure 3: **Time courses for the most- and least-fair subsidisation schemes when the tolerant and resistant crop are inefficient.** (A) Dynamics that maximise the profits to the grower. Here, ≈ 49.8% of growers use resistant crop. (B) The fairest scenario, where both the profits and costs have relatively equal weighting. In this case, ≈ 40.3% use resistant crop. (C) When minimising the cost to the planner is prioritised, ≈ 10% of growers use tolerant crop.

11.3 Effect of change in cost of improved crop on the proportion of tolerant or resistant crops

When the cost of resistant (ϕ_R) and tolerant (ϕ_T) crops are varied, there is only a narrow parameter space where both crop types can coexist (Figure 6(D) in the main text). For the majority of the parameter space, two distinct regions emerge: one where growers use resistant and unimproved crop, and one where growers use tolerant and unimproved crop. The Pareto front lies within the "resistant and unimproved" equilibrium, save for the solution at $\phi_T = 0.375$ and $\phi_R = 0.4$, where no growers use

resistant crop and 10% use tolerant crop.

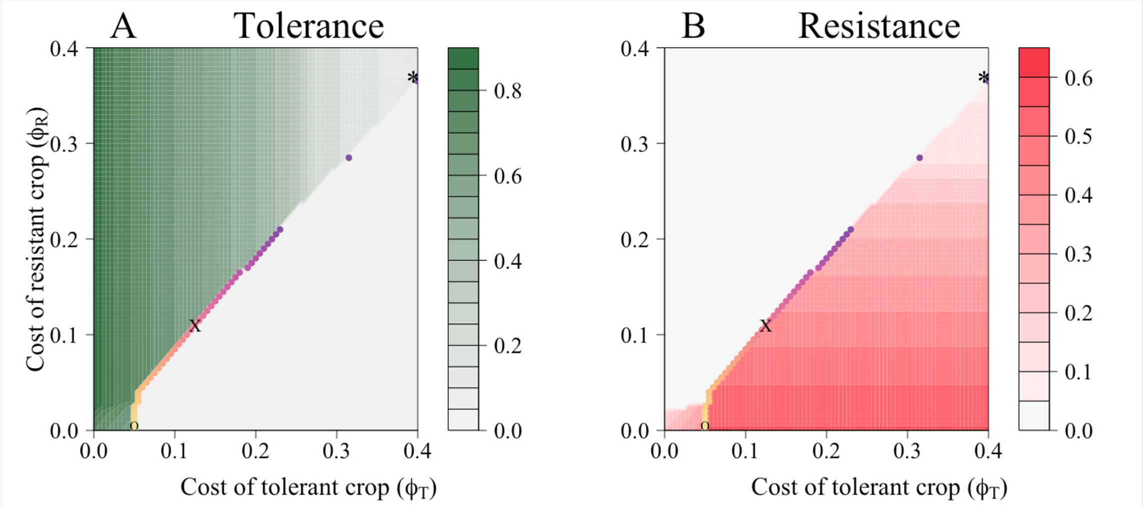

Figure 4: **Effect of change in the price of tolerant (ϕ_T) and resistant (ϕ_R) crop on the proportion of growers using improved crop over time.** Change in uptake of (A) tolerant crop and (B) resistant crop. Along the Pareto front, growers are always in a "resistant and unimproved" equilibrium, except for the point at $\phi_T = 0.375$ and $\phi_R = 0.4$, where no growers use resistant crop and 10% of growers use tolerant crop. The fairest scenario is marked with a "X" (Gini coefficient = 0.009, when $\tau = 0.13$ and $P = 0.93$). The least fair scenarios ($G = 0.5$) are marked with "O" when the profit is prioritised, and "*" when the costs to the planner are.
